# Multimodal Integration of Protein Interactomes With Genomic and Molecular Data Discovers Distinct Rheumatoid Arthritis Endotypes

**DOI:** 10.1002/art.70091

**Published:** 2026-03-09

**Authors:** Javad Rahimikollu, Priyamvada Guha Roy, Akash Kishore, Danica Morgan Lee, Lauren A. Vanderlinden, Kiran Nazarali, Fan Zhang, Dana P. Ascherman, Daniella M. Schwartz, Larry Moreland, Jishnu Das

**Affiliations:** ^1^ Center for Systems Immunology Department of Immunology, University of Pittsburgh Pittsburgh Pennsylvania; ^2^ Department of Computational and Systems Biology University of Pittsburgh Pennsylvania; ^3^ Joint Carnegie Mellon University‐University of Pittsburgh PhD Program in Computational Biology Pittsburgh Pennsylvania; ^4^ Department of Human Genetics School of Public Health, University of Pittsburgh Pittsburgh Pennsylvania; ^5^ Division of Rheumatology and Clinical Immunology, Department of Medicine University of Pittsburgh School of Medicine Pittsburgh Pennsylvania; ^6^ Division of Rheumatology University of Colorado Denver Aurora Colorado

## Abstract

**Objective:**

Rheumatoid arthritis (RA) is a heterogeneous autoimmune disease characterized by clinical and molecular heterogeneity, notably in the presence of anti–cyclic citrullinated peptide (CCP) antibodies. Patients with CCP+ RA exhibit more severe disease progression and distinct treatment responses compared to patients with CCP− RA. Although previous studies have investigated cellular and molecular differences between these subtypes, their genetic differences are understudied.

**Methods:**

We leveraged the Rheumatoid Arthritis Comparative Effectiveness Research cohort, comprising 555 patients with CCP+/rheumatoid factor (RF)+ RA and 384 patients with CCP−/RF+ RA. Using a novel framework, we integrated a network‐based genome‐wide association study (GWAS) with multiomic data to uncover corresponding genetic and molecular differences.

**Results:**

We uncovered a significant heritability difference between these disease groups. Network‐based GWAS uncovered 14 putative gene modules, including many genes outside the HLA loci, that explained genetic differences between CCP+/RF+ and CCP−/RF+ RA. Heritability partitioning and multivariate expression analyses validated four modules, highlighting novel genetic loci underlying phenotypic differences. Module functional significance was established using multiple orthogonal cohorts, underscoring their biologic relevance.

**Conclusion:**

Our findings demonstrate the use of network‐based approaches in revealing differential genetic risk factors underlying CCP+/RF+ and CCP−/RF+ RA. Disease‐associated gene modules detected in synovial tissue were also observed in peripheral blood, indicating joint‐specific molecular programs are reflected systemically. This cross‐tissue concordance highlights the potential for blood‐based assays to capture pathogenic mechanisms active in the joints, enabling practical patient stratification. Our findings highlight why patients with CCP+/RF+ and CCP−/RF+ RA exhibit distinct clinical courses and therapeutic responses, supporting precision‐guided treatment strategy development in RA.

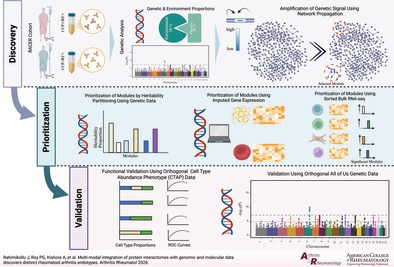

## Graphical abstract

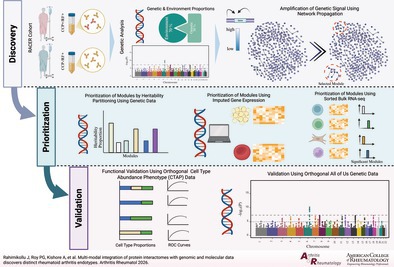

## INTRODUCTION

Rheumatoid arthritis (RA) is a chronic, progressive autoimmune disease characterized by inflammation of synovial joints and systemic immune dysregulation. According to the Global Burden of Diseases, Injuries, and Risk Factors Study 2021, approximately 17.6 million people worldwide are affected by RA.^1^ Beyond its clinical manifestations, RA exhibits considerable molecular and cellular heterogeneity, which contributes to variations in disease onset, progression, and treatment response. A key source of this heterogeneity lies in the anti–cyclic citrullinated peptide (CCP) antibodies and rheumatoid factor (RF) in the serum. Although these autoantibodies form part of the diagnostic criteria for RA, many patients are seronegative for one or both.^2^

The serological profile of these autoantibodies defines two major subgroups: CCP+/RF+ (doubly seropositive) and CCP–/RF+ (singly seropositive) RA. Although CCP seropositivity has been associated with a more aggressive disease course and a higher risk of joint destruction,^3^ some studies suggest that patients with CCP+ RA may respond more favorably to certain disease‐modifying antirheumatic drugs.^4^ Despite these clinical differences, both subgroups are treated with the same therapeutic interventions, reflecting the assumption that they share similar pathogenic mechanisms. However, emerging molecular evidence suggests that these subgroups may represent distinct biologic endotypes rather than variations of a single disease.^1^

Previous studies have reported immunologic and transcriptomic differences between CCP+/RF+ and CCP−/RF+ RA,^5^, ^6^, ^7^ yet their genetic basis remains poorly understood. Comparisons of findings from case‐control studies for CCP+/RF+ and CCP−/RF+ RA have shown that both disease endotypes have shared and distinct genetic loci underlying their pathogenic architectures.^8^, ^9^, ^10^ However, there have been limited efforts directly comparing them.^11^ Variants that distinguish RA cases from healthy controls are concentrated within the HLA region and reflect general autoimmune susceptibility; further, most studies have considered either disease subtype but not both or have grouped them together.^12^, ^13^, ^14^, ^15^, ^16^ However, whether genetic differences separating CCP+/RF+ and CCP−/RF+ RA arise from the same broad pathways that drive RA versus control differences or distinct processes has not been systematically explored. Addressing this gap is essential for defining the specificity of endotype‐associated mechanisms and for identifying genetic programs that shape disease heterogeneity beyond overall autoimmunity.

Genome‐wide association studies (GWASs) involve testing differences in allele frequency to find associations between genetic variants and phenotypes.^17^ Although GWASs remain a cornerstone of genetic research, they have several limitations.^18^ Importantly, the statistical power for detecting associations depends on the sample size of the cohort. This is especially limiting when studying disease subsets with small cohort sizes. Given that increasing sample size to facilitate discovery of novel genetic loci is not always feasible, other methods are needed to improve discovery. One such method is network‐based GWAS. It involves leveraging biologic networks, such as protein–protein interaction (PPI) networks, because interacting proteins usually are involved in the same molecular pathways and are often implicated in the pathogenesis of the same trait.^19^ By propagating the signal from a GWAS over the PPI network, it is possible to augment signal by borrowing strength from functionally similar genes, which were not significantly associated with the trait due to low power, and thus identify additional disease‐associated loci.^20^ Although network‐based analyses have provided mechanistic insights in other autoimmune conditions, such as multiple sclerosis^21^ and Crohn disease,^22^ they have rarely been applied to distinguish within‐disease heterogeneity. Applying such a framework to characterize patients with RA defined by differences in CCP and RF serology provides a unique opportunity to reveal whether their genetic architectures represent extensions of the same disease spectrum or reflect fundamentally distinct pathophysiology.

This study employs a multitiered analytical framework integrating genomic and transcriptomic data to uncover molecular architecture distinguishing CCP+/RF+ and CCP−/RF+ endotypes. Although conventional GWASs assume independence among genetic loci and identify individual loci associated with phenotype, they often fail to reveal how these variants converge into functional biologic processes, leaving a gap between genetic associations and clinical manifestation. To bridge this gap, we first used genetic data to assess differences in heritability, providing an indication of difference in genetic architectures between the two endotypes. Partitioning heritability across gene modules demonstrated the prioritized modules capture a substantial proportion of endotype‐specific genetic variance. Integration of transcriptomic data and multiomics analysis further refined these modules by adding a layer of cell‐type and tissue specificity, linking heritable variation to gene expression programs and cellular phenotypes. Together, this integration provides mechanistic insight into how genetic risk manifests in tissue‐specific and cell‐type–specific contexts.

## METHODS

### Cohort description

In this study, we used data from the University of Pittsburgh Rheumatoid Arthritis Comparative Effectiveness Research (RACER) registry, which consisted of prospectively enrolled patients with RA recruited through the University of Pittsburgh Medical Center. The cohort included 555 and 384 patients with CCP+/RF+ and CCP−/RF+ RA, respectively. Genotype data was collected for all 939 patients using the MegaChip array. We filtered subjects based on the quality of raw genotypes as well as relatedness to exclude highly related subjects (kinship value >0.12 = same family) because they are likely to introduce biases. A total of 7,173,418 single nucleotide polymorphisms (SNPs) were imputed using the Sanger imputation server.^23^ This study was approved by the University of Pittsburgh Institutional Review Board (protocol 19090282) and adhered to the principles outlined in the Declaration of Helsinki.

### Genome‐wide association analyses adjusting for appropriate covariates

Phenotypes were encoded in a vector *Y* of length 939, with values of 1 for CCP+/RF+ and 0 for CCP−/RF+ RA. Genotype data comprised 7,173,418 SNPs per individual and were organized into a 939 × 7,173,418 matrix (*X*, with rows representing individuals and columns representing SNPs. To adjust for confounding, we included biologic sex, age, and the first five genetic principal components as covariates. Genotype quality control was performed using PLINK,^24^ applying filters for SNP and sample missingness (≤1%) and minor allele frequency (≥5%). Conditional analyses were conducted using the COJO tool in Genome‐Wide Complex Trait Analysis (GCTA).^25^ Primary SNPs were first identified via stepwise selection (–cojo‐slct), followed by conditional GWAS (–cojo‐cond) across the significant HLA region.

### Heritability estimation using LDAK

We used the LDAK^26^ model to estimate heritability of the phenotypic trait. To determine the difference in heritability, we began by computing the genetic relatedness matrix (GRM) using the ldak5.2 linux executable obtained from the LDAK website. After obtaining the GRM, we applied the –reml option to calculate heritability differences.^27^ Details of the heritability estimation are provided in the Supplementary Notes.

### Network propagation using random walk with restart using gene‐centric scores

In this study, we applied the HotNet2 algorithm to conduct network propagation analyses on the reference human PPI network.^28^ Additional details are explained in the Supplementary Notes.

### Heat diffusion and module detection in network propagation to uncover functionally coherent subnetworks

Gene‐level relevance was quantified by assigning each gene a heat score defined as the −log(*P* value) from restricted maximum likelihood (REML) LDAK heritability estimates for SNPs within 50 kb, encoding endotype status (doubly seropositive = 1; singly seropositive = 0), propagating these scores across a PPI network using a random walk with restart algorithm,^29^ and identifying statistically significant subnetworks via 500 degree‐preserving network permutations, yielding 14 validated modules comprising 4 to 54 genes.

### Heritability partitioning using LDAK: module‐specific kinship matrix calculation and REML analysis

Heritability was partitioned across 14 gene modules using the LDAK tool by constructing module‐specific kinship matrices (–calc‐kins‐direct,–power −0.25) and estimating module‐level heritability via REML while adjusting for sex and principal components (Supplementary Notes).

### Imputation of gene expression using PrediXcan for tissue‐specific analysis of discriminatory gene networks

We used PrediXcan^30^ to impute tissue‐specific gene expression from genotypes using linear allele–expression models. Expression was imputed for whole blood and subcutaneous adipose tissue, relevant to RA. Linear regression tested associations with CCP+ versus CCP− RA, and −log10(*P* value) quantified tissue‐specific network module discrimination across RA endotypes and network modules analysis (Supplementary Notes).

### Evaluation of gene module discriminatory performance using bulk RNA‐sequencing data

Bulk RNA‐sequencing (RNA‐seq) data from sorted synovial T cells, B cells, monocytes, and fibroblasts in 29 Accelerating Medicines Partnership RA phase 1 patients^31^ with available CCP/RF serostatus were obtained from ImmPort (SDY998), filtered for low‐variability genes, and analyzed using least absolute shrinkage and selection operator (LASSO)–regularized logistic regression in R to evaluate the discriminatory power of network‐derived gene modules, with model training and performance assessed by leave‐one‐out cross‐validation and area under the receiver operating characteristic (ROC) curve (AUC)–based tuning. Additional details are in the Supplementary Notes.

### Replication GWAS using All of Us cohort

As a replication cohort, we analyzed 248 RF+ participants of European ancestry from the All of Us Research Program,^32^ stratified into CCP+/RF+ (n = 93) and CCP−/RF+ (n = 155), with phenotype encoded as a binary vector and genotype data comprising 6,879,629 SNPs, which were quality controlled using PLINK with genotype missingness (10%) and minor allele frequency (5%) filters. Additional details are in the Supplementary Notes.

### Evaluation of gene module performance in cell‐type abundance phenotype and treatment response classification using single‐cell RNA‐seq data

Using single‐cell RNA‐seq (scRNA‐seq) data from 70 patients with RA from the Accelerating Medicines Partnership RA phase 2 cohort,^33^ we generated pseudobulk profiles across immune and stromal cell types and applied LASSO‐regularized logistic regression in a one‐versus‐rest framework to evaluate the predictive performance of network‐derived gene modules for cell‐type abundance phenotype (CTAP) and treatment response classification, with model training, cross‐validation, and ROC‐AUC–based performance assessment conducted in R using glmnet, caret, pROC, and dplyr. Additional details are in the Supplementary Notes.

### Code and data availability

All data and code associated with the manuscript are available at https://github.com/jishnu-lab/RAEndo.

## RESULTS

**Figure 1:**
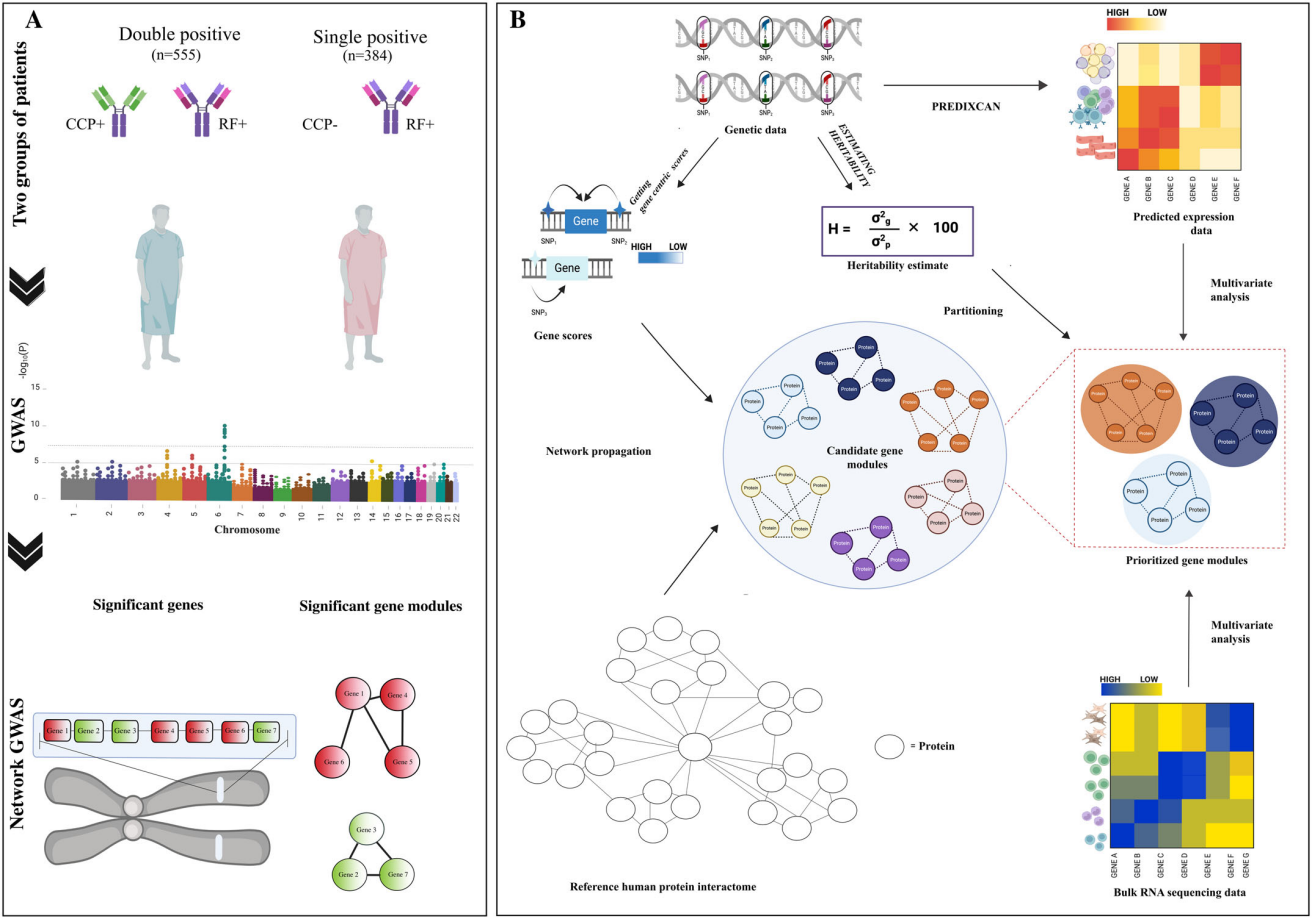
Multiscale network‐informed genetic analyses to identify heritability and phenotypic differences across CCP+/RF+ and CCP−/RF+ rheumatoid arthritis. (A) Conceptual overview of our study. (B) Schematic representation of analytical pipeline used to identify and validate genetic loci underlying phenotypic differences between CCP+/RF+ and CCP−/RF+ rheumatoid arthritis. CCP, anti–cyclic citrullinated peptide; GWAS, genome‐wide association study; RF, rheumatoid factor; SNP, single nucleotide polymorphism.

We leveraged the RACER cohort,^34^ which comprised 555 and 384 patients with CCP+/RF+ and CCP−/RF+ RA, respectively (Figure 1A), predominantly of White ancestry. We looked to elucidate genetic and molecular differences between these subtypes of RA. Because a conventional GWAS would not be sufficiently powered at these sample sizes to elucidate differences between these disease subtypes, we employed a network‐based GWAS approach. By considering the interactions and relationships among genes, a network‐based GWAS can identify genetic associations that may be missed by traditional single‐variant analysis (Figure 1B). Using this method, we could prioritize clusters of genes or gene modules that can shed light on functionally relevant pathways. These modules underwent further orthogonal analyses to establish biologic significance.

### Heritability analysis highlights distinct genetic underpinnings of CCP+/RF+ and CCP−/RF+ RA, but conventional GWAS implicates only HLA loci

**Figure 2:**
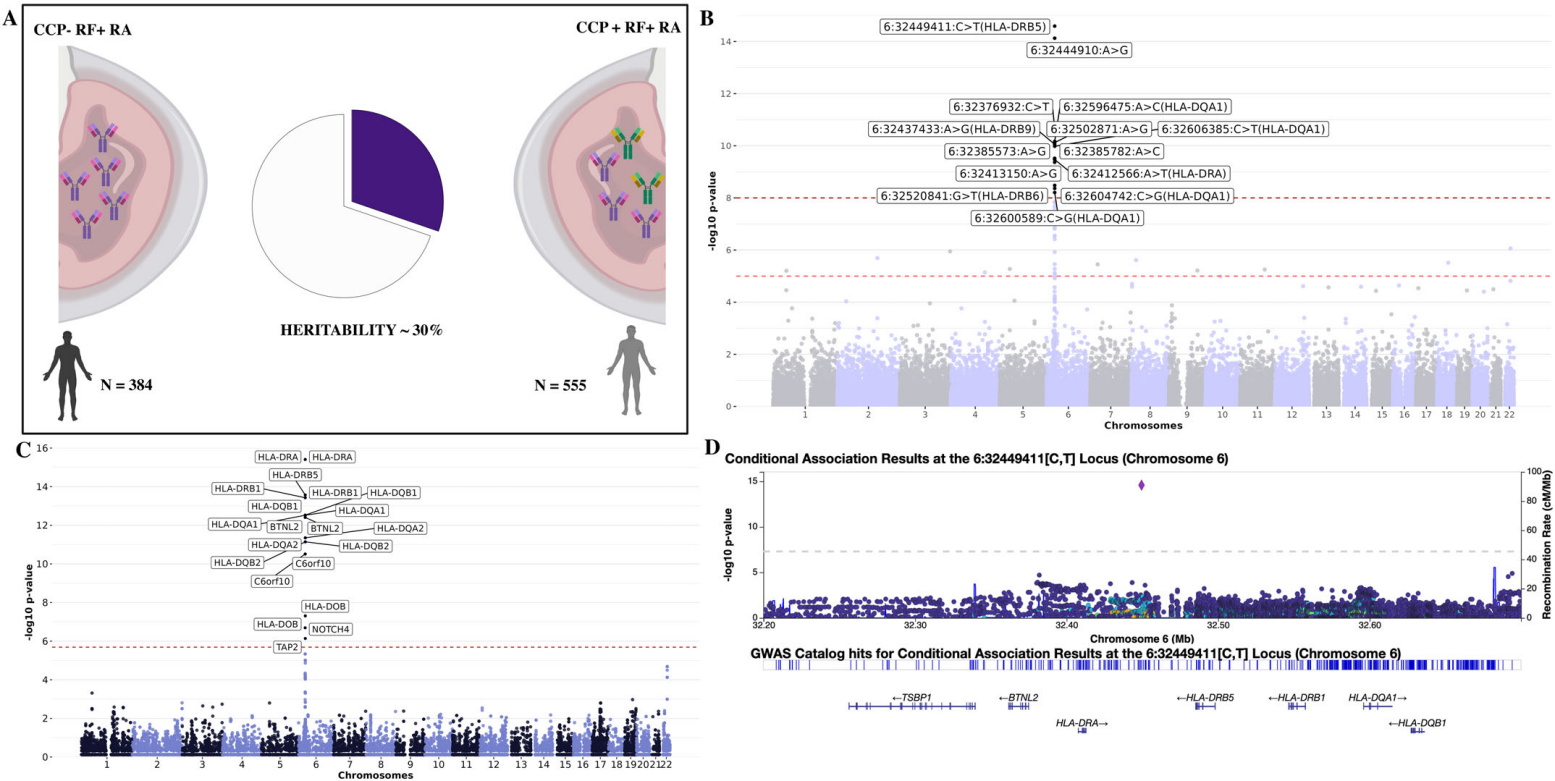
Genetic loci identified using conventional GWAS do not explain the difference in heritability between CCP+/RF+ and CCP−/RF+ RA. (A) Difference in heritability between CCP+/RF+ and CCP−/RF+ RA within the Rheumatoid Arthritis Comparative Effectiveness Research cohort. (B) Manhattan plot for GWAS, corrected for covariates with one principal component and biologic sex, comparing CCP+/RF+ and CCP−/RF+ RA in which single nucleotide polymorphisms with significant association statistics are labeled. (C) Manhattan plot for gene‐based association analyses, corrected for covariates with one principal component and biologic sex, comparing CCP+/RF+ and CCP−/RF+ RA in which genes with significant association statistics are labeled. CCP, anti–cyclic citrullinated peptide; GWAS, genome‐wide association study; RA, rheumatoid arthritis; RF, rheumatoid factor.

To quantify genetic differences between CCP+/RF+ and CCP−/RF+ RA, we estimated subgroup‐specific heritability using genotypes from 939 RACER participants. After excluding variants with minor allele frequency <0.05, approximately 7.2 million SNPs were analyzed, and heritability differences were estimated using the LDAK REML approach.^10^ Given the female predominance (women:men 3.5:1) and the known role of age in RA risk, we evaluated two fixed‐effect models: (1) adjusting for one principal component and sex (Figure 2A) and (2) adjusting for five principal components, sex, and age. Ages were similar between patients with CCP+/RF+ (60 years) and CCP−/RF+ RA (Supplementary Figure 1A). Both models yielded a heritability difference of 0.30 (*P* = 0.03; Figure 2A), indicating that approximately 30% of phenotypic differences are genetically driven and supporting distinct endotypes. These results align with a Swedish familial aggregation study reporting heritability of approximately 50% for CCP+/RF+ RA and approximately 20% for CCP−/RF+ RA^35^ but contrast with findings from a prior twin study.^36^

To identify which genetic loci underlie these differences, we conducted a GWAS using two covariate models: (1) adjusting for one principal component and sex (Figure 2B) and (2) adjusting for five principal components, sex, and age (Supplementary Figures 1B and 1C). The results were comparable across both models, indicating that the first covariate setting was sufficient. Our analysis identified 2,441 genetic variants with associated *P* values meeting the Bonferroni‐corrected significance threshold of 5 × 10^−8^. All the genome‐wide significant variants were confined to the HLA region on chromosome 6, and a handful of additional variants surpassed a nominal *P* value threshold of 5 × 10^−5^ (Figure 2B).

To dissect association signals within the HLA region, we performed stepwise regression followed by conditional analyses using GCTA‐COJO,^25^ which identified *HLA‐DRB5* as the primary driver of the strong HLA association. The lead variant 6:32449411:C>T captured the core genetic effect and markedly attenuated residual signals after conditioning (Figure 2C). Although this finding is consistent with the central role of HLA class II alleles in RA susceptibility^37^ and prior GWAS comparing CCP+/RF+ and CCP−/RF+ RA cases, significant GWAS SNPs explained only about 3% (ie, only 10% of the ~30% heritability difference between the two groups), indicating substantial false negatives due to stringent false discovery rate control (Figure 2B). Univariate gene‐based association tests using LDAK identified significant associations with *HLA‐DRB1*, *HLA‐DRB5*, and *HLA‐DRA* within the HLA region (Figure 2B) but remained largely locus confined and are inherently underpowered because they do not account for intergene relationships or joint effects (Figure 2D).

### Network GWAS unveils additional loci underlying the phenotypic differences between CCP+/RF+ and CCP−/RF+ RA

With this cohort size, a conventional GWAS was underpowered to detect associations beyond the HLA locus. To address this, we adopted a network‐based GWAS approach that integrates genetic association data with biologic networks to identify genes implicated for a given phenotype. High‐scoring genes that are randomly distributed across the network may not reflect meaningful biologic associations. In contrast, moderately scoring genes that are highly proximal within the network are more likely to indicate a real signal because they often participate in the same pathways and contribute to the same traits. This approach is based on the premise that interacting proteins usually operate in similar molecular pathways and are commonly linked to the same trait.^20^

**Figure 3:**
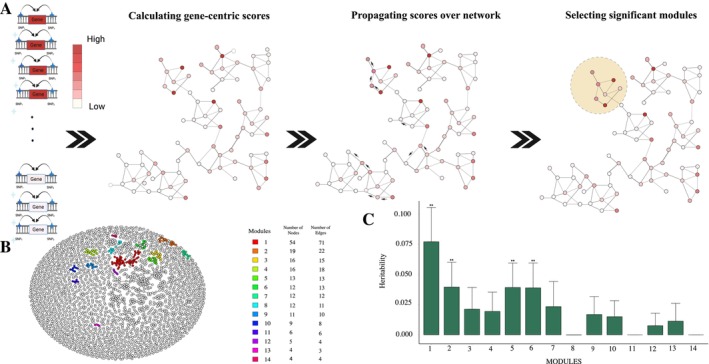
Network genome‐wide association study identifies additional associations underlying genotypic differences between CCP+/RF+ and CCP−/RF+ RA. (A) Schematic representation of how network propagation was used to identify genetic loci underlying the phenotypic differences between CCP+/RF+ and CCP−/RF+ RA. (B) Network‐based genome‐wide association study analysis identifies 14 significant modules as candidates explaining genotypic and heritability differences between CCP+/RF+ and CCP−/RF+ RA. (C) Bar plot showing per‐module contribution to the difference in heritability between CCP+/RF+ and CCP−/RF+ RA, where ** represents significant contributions as measured by the LDAK restricted maximum likelihood *P* value. CCP, anti–cyclic citrullinated peptide; RA, rheumatoid arthritis; RF, rheumatoid factor; SNP, single nucleotide polymorphism.

We used LDAK REML to estimate a comprehensive gene‐centric score that account for coding variants and noncoding variants that modulate gene expression (Figure 3A), accounting for the underlying linkage disequilibrium structure. We then propagated these scores over a high‐quality reference protein interactome^28^ using a random walk with restart algorithm.^29^ Critically, the network used here reflects connectivity at the functional level between proteins encoded by these genes and not at the locus level. The incorporation of multiscale (genetic and functional associations) and multiomic (genomic and interactome) data enables the identification of gene modules missed by a conventional GWAS. To determine both the number and size of significant gene modules, we applied a permutation‐based statistical test (Supplementary Notes). Using this approach, we identified 14 statistically significant network modules (Supplementary Figure 2). These modules comprised 193 genes (Figure 3B), including genes from the HLA region and a wide array of other molecular pathways, which have not been previously implicated in the phenotypic differences between CCP+/RF+ and CCP−/RF+ RA.

Our analyses identified modules that significantly correlate with phenotypic differences between CCP+/RF+ and CCP−/RF+ RA. To move beyond correlations, we interrogated these modules in terms of their ability to explain heritability partitioning as well as against several functional genomic data sets. This multistep prioritization system helped identify the functional significance of the modules using multiple orthogonal analyses and data sets. The use of a combination of multiple layers of evidence (genetic, cellular, and molecular) from different data sets helped us converge on the most robust and biologically meaningful modules that explain heterogeneity between these two subtypes of RA.

### Candidate gene modules explain the difference in heritability

To assess the relative importance of each gene module, we estimated module‐specific heritability contributions using LDAK REML. Conventional GWAS SNPs, limited to the HLA locus, explained only approximately 3% (~10% of the ~30%) of the heritability difference between subgroups. In contrast, combining heritability across network modules recapitulated an approximately 31% difference, comparable to genome‐wide estimates, enabling prioritization of modules by genetic contribution. Beyond the HLA module (module 1; h^2^ = 0.075), modules 2, 5, and 6 showed significant independent contributions (Figure 3C).

### Multivariate analysis using sorted bulk RNA‐seq data reveals cell type–specific signals for candidate gene modules

In addition to explaining heritability, we hypothesized that functionally important network modules would correspond to genes whose expression programs are significantly different between doubly and singly seropositive RA. So, we performed an orthogonal validation analysis using a data set of bulk RNA‐seq from the Accelerating Medicines Partnership RA phase I data.^31^ Interestingly, although this cohort included patients with CCP+/RF+ RA, most of the singly seropositive patients were CCP+/RF− and a few were CCP−/RF+. However, in the RACER cohort, the singly seropositive patients were CCP−/RF+. Despite this difference, the analysis allows us to evaluate whether the identified modules are overall discriminatory between doubly and singly seropositive RA.

**Figure 4:**
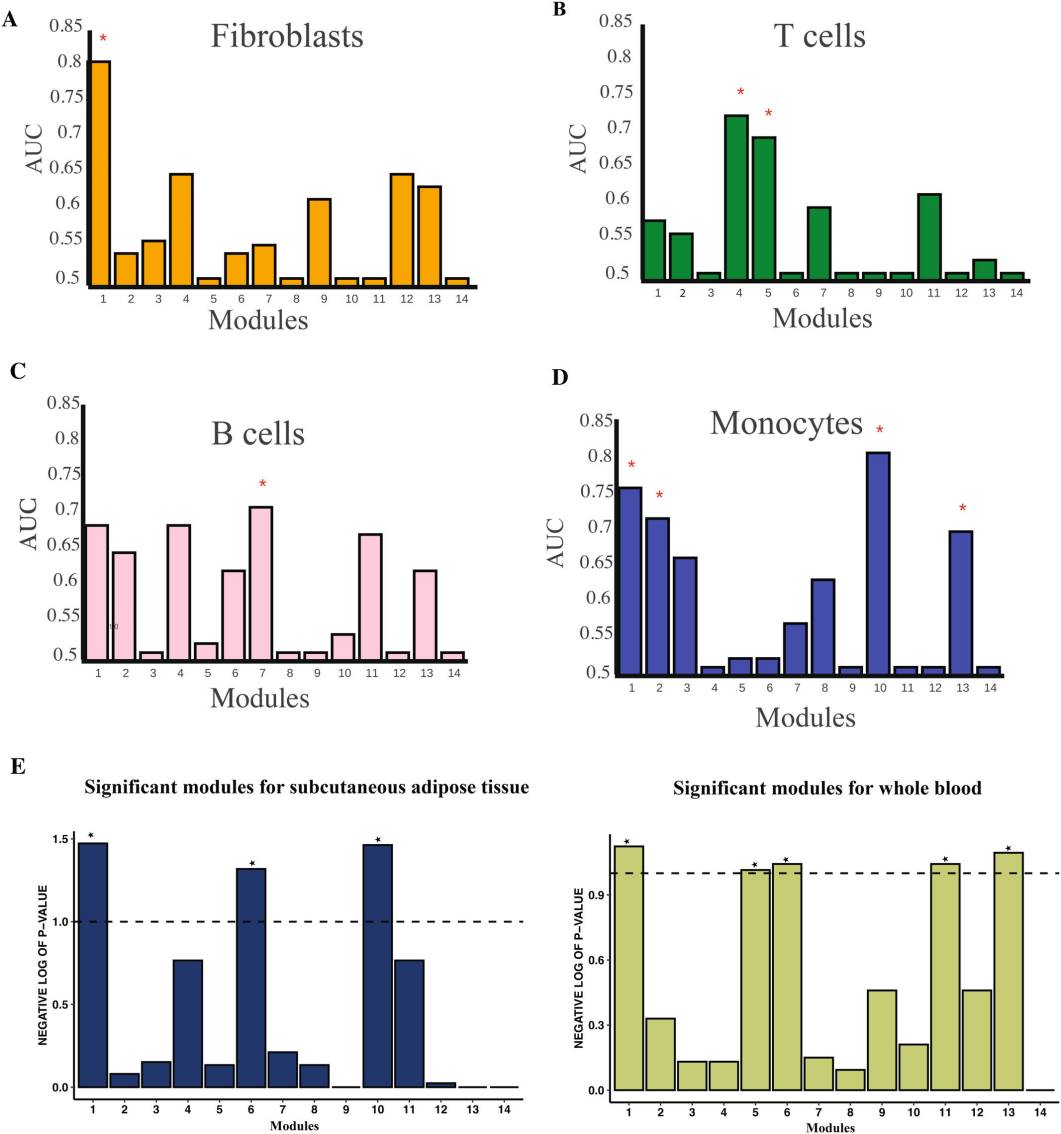
Multivariate analyses of sorted bulk RNA‐sequencing data and imputed gene expression data reveal cell type and tissue specificity of candidate gene modules. (A–D) Bar plot showing the discriminative ability of a multivariate logistic regression model based on gene expression profiles for each module in fibroblasts (A), T cells (B), B cells (C), and monocytes (D), where AUC values represent the model's ability to discriminate anti–cyclic citrullinated peptide+/rheumatoid factor+ rheumatoid arthritis from other rheumatoid arthritis subsets and * represents the model's performance against a score distribution‐matched module. (E) Bar plot showing the discriminative ability of a multivariate logistic regression model based on predicted gene expression for each module in subcutaneous adipose tissue, where −log(*P* values) represent the discriminatory power of the modules and * represents models with −log(*P* value) ≥ 1. AUC, area under the receiver operating characteristic curve.

We tested whether the expression of genes encoding proteins within the identified network modules could significantly discriminate CCP+/RF+ RA from other subtypes across different cell types, using AUC as the performance metric. We performed permutation testing by randomly selecting size‐matched gene sets from all genes in the bulk RNA‐seq data. In fibroblasts, the interferon signaling and extracellular matrix remodeling (ECM) module (module 1) was discriminatory between doubly versus singly seropositive RA (Figure 4A). Genes coding for proteins comprising the immune response and inflammation modulation and complement system activation and regulation modules (modules 4 and 5, respectively) were found to be discriminatory in T cells (Figure 4B). The cytokine signaling and immune response modulation module (module 7) was significant in B cells (Figure 4C), and the interferon signaling and ECM, TGF‐β/BMP signaling in developmental processes, protein folding and glycosylation in the endoplasmic reticulum, and chemokine‐mediated signaling in immune response modules (modules 1, 2, 10, and 13, respectively) were significant in monocytes (Figure 4D).

### Multivariate analysis using imputed expression from RACER cohort prioritizes specific candidate gene modules

To further validate our findings at the expression level, we imputed the expression levels of genes in the 14 modules for subjects in the RACER cohort based on their individual genotypes. We used PrediXcan,^30^ a regularized regression approach that leverages individual genetic variation to predict corresponding expression levels for each subject. This is an orthogonal but equally important approach because we used imputed matched (at a per‐subject level) gene expression in this analysis as opposed to an orthogonal cohort in the prior analysis. By using the PrediXcan model, we were able to impute expression levels for both adipose tissue and whole blood. Consistent with the prior transcriptomic analysis using data from the AMP cohort, this assessment revealed that several of the 14 identified modules were again significantly discriminatory between CCP+/RF+ and CCP−/RF+ RA in adipose tissue and whole blood (Figure 4E).

### Network GWAS identifies cell type–specific modules that stratify disease endotypes

**Figure 5:**
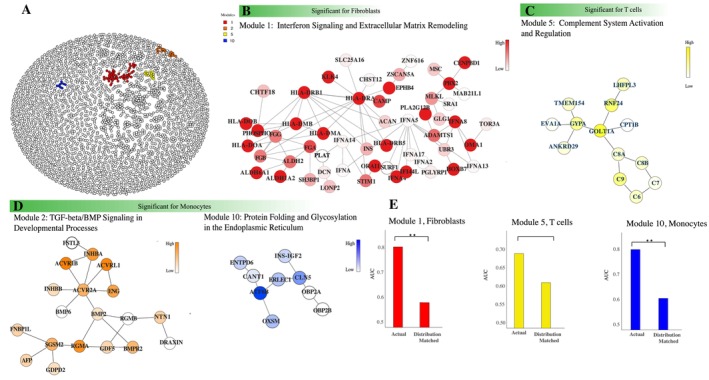
Prioritized gene modules reveal novel cross‐talk between HLA–type 1 interferon underlies the phenotypic differences seen between anti–cyclic citrullinated peptide+/rheumatoid factor+ and other rheumatoid arthritis subsets. (A) Visualization of prioritized gene modules in the overall network (prioritized modules are colored). (B) Module with significant performance in multivariate analysis using expression data from fibroblasts. (C) Module with significant performance in multivariate analysis using expression data from T cells. (D) Module with significant performance in multivariate analysis using expression data from monocytes. (E) Gene modules showing significantly higher discriminatory power compared to random distribution‐matched gene sets. AUC, area under the receiver operating characteristic curve.

Our study introduced a robust and innovative method for identifying genetic factors underlying the phenotypic difference between patients with CCP+/RF+ and CCP−/RF+ RA. Using a network‐based GWAS approach, we identified 14 candidate gene modules (Figure 5A). Five of these modules, interferon signaling and ECM (module 1, Figure 5B), complement system activation and regulation (module 5, Figure 5C), TGF‐β/BMP signaling in developmental processes (module 2, Figure 5D), protein folding and glycosylation in the endoplasmic reticulum (module 10, Figure 5D), and protein processing and cellular transport (module 6, Supplementary Figure 2), successfully passed two of the three stringent validation analyses.

We were able to assign cell‐type specificity for four of the five modules, and we consider these the final validated modules for downstream functional analyses and biologic interpretation using a large‐language model–based approach^38^ (Figure 5B–5D). We were unable to specify cell‐type specificity for one module, which may be interesting for future follow‐up (module 6, Supplementary Figure 2). Together, these modules comprise 107 genes. The largest module, comprising 54 genes, included genes from within the HLA region and other immune signaling genes. The other modules encompassed a wide range of biologic processes. Despite the vast search space of >17,000 proteins in the entire network, our method demonstrates a high level of specificity (Figure 5A). Interestingly, these modules contain genes with both high and low gene scores, highlighting the potential of our approach to identify genes that may be overlooked in traditional GWAS analyses.

Our analysis identified a significant module comprising complement system genes, including *C9*, *C8A*, *C8B*, *C7*, and *C6* (Figure 5C). The complement system is a crucial part of the innate immune response, and its dysregulation has been implicated in autoimmune diseases such as RA. Notably, the presence of complement genes encoding components of the membrane attack complex in module 5 (complement system activation and regulation) indicates a potential involvement of complement‐mediated cytotoxicity and subsequent tissue damage mechanisms in RA (Figure 5C). Overall, our findings shed new light on the complex interplay of genes and proteins in the mechanism of RA and open new avenues for further research and potential therapeutic interventions.

Gene modules represent structured subnetworks rather than merely groups of unrelated genes. To highlight the importance of this connectivity structure within our identified modules, we compared the performance of candidate gene modules against “distribution‐matched” control modules (Supplementary Notes). Permutation testing against random size‐matched modules showed that for two of the four modules, addition of connectivity structure improved discriminatory power (AUC) beyond gene membership alone, whereas for the other two modules, gene membership alone was sufficient (Figure 5E). Even with this stringent test, we observed significant differences in the predictive power of our modules compared to the “distribution‐matched, size‐matched” controls. Specifically, these differences were notable for the interferon signaling and ECM module (module 1) in T cells and the protein folding and glycosylation in the endoplasmic reticulum module (module 10) in monocytes. Together, these results indicate that the identified modules underlie key phenotypic differences in doubly versus singly seropositive RA.

### Validation of selected gene modules using orthogonal genetic data from the All of Us cohort

**Figure 6:**
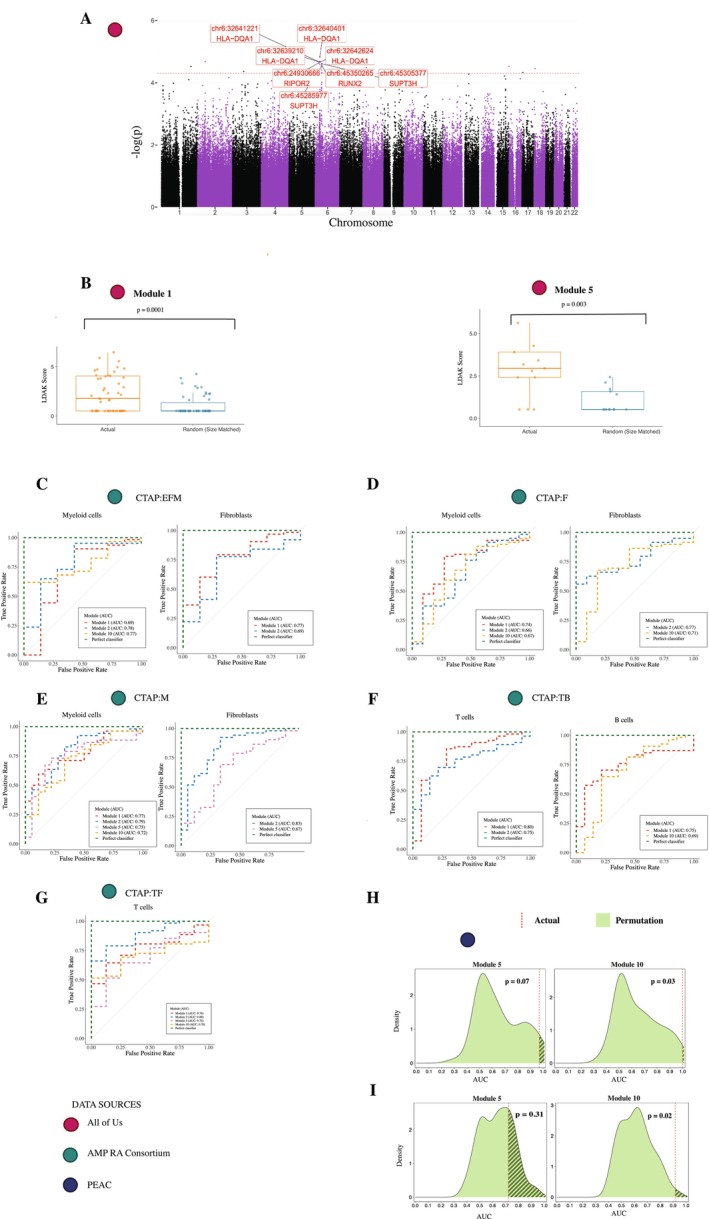
Functional validation of CCP‐associated gene modules. (A) Manhattan plot for genome‐wide association study comparing CCP+/RF+ and CCP−/RF+ RA in the All of Us cohort. (B) Comparison of LDAK scores for prioritized gene modules versus size‐matched random gene sets in the All of Us cohort. (C) ROC curves evaluating the ability of gene modules to distinguish CTAP‐EFM from other CTAP phenotypes using scRNA‐seq data. (D) ROC curves evaluating the ability of gene modules to distinguish the CTAP‐F from other CTAP phenotypes using scRNA‐seq data. (E) ROC curves evaluating the ability of gene modules to distinguish the CTAP‐M from other CTAP phenotypes using scRNA‐seq data. (F) ROC curves evaluating the ability of gene modules to distinguish the CTAP‐TB from other CTAP phenotypes using scRNA‐seq data. (G) ROC curves evaluating the ability of gene modules to distinguish the CTAP‐TF from other CTAP phenotypes using scRNA‐seq data. (H) Distribution of AUC values from 10‐fold cross‐validation distinguishing CCP+/RF+ from CCP−/RF+ RA. The null distribution was obtained by permutation testing, wherein seropositivity labels were randomly shuffled. (I) Distribution of AUC values from 10‐fold cross‐validation distinguishing patients with RF+ from RF− RA. The null distribution was obtained by permutation testing, wherein group labels (CCP & RF status) were randomly shuffled. AMP, Accelerating Medicines Partnership; AUC, area under the receiver operating characteristic curve; CCP, anti–cyclic citrullinated peptide; CTAP, cell‐type abundance phenotype; PEAC, Pathobiology of Early Arthritis cohort; RA, rheumatoid arthritis; RF, rheumatoid factor; ROC, receiver operating characteristic; scRNA‐seq, single‐cell RNA‐sequencing.

To independently validate the gene modules identified from network GWAS of the RACER cohort, we leveraged genotype and phenotype data from the All of Us cohort.^32^ We restricted our analysis to European ancestry individuals who were RF+ (RF > 20 IU/mL), yielding a cohort of 248 patients with RA, a population composition like that of the RACER cohort. These samples were further stratified based on anti‐CCP antibody status into double‐positive (CCP+/RF+; n = 93) and single‐positive (CCP−/RF+; n = 155) subgroups, with CCP values >20 IU/mL considered positive (Supplementary Figure 3A). A GWAS comparing these two groups revealed significant enrichment at the HLA class II locus on chromosome 6 (Figure 6A), consistent with findings in the RACER cohort (Figure 2B). We used LDAK to aggregate SNP scores for each gene and compared the scores of genes from our modules to distributions derived from random, size‐matched gene sets. Notably, two modules—interferon signaling and ECM (module 1) and complement system activation and regulation (module 5)—showed significantly higher genetic scores relative to random expectations (Figure 6B), highlighting the robustness of our findings and the importance of these genes in shaping the phenotypic differences between the endotypes.

### Prioritized gene modules capture molecular processes that explain heterogeneity in RA beyond seroprevalence of autoantibodies

Beyond serotype‐based heterogeneity, patients with RA also display additional clinical variation, one aspect of which is captured by CTAPs.^33^ CTAPs reflect distinct synovial inflammatory microenvironments, ranging from lymphocyte rich (CTAP‐TB and CTAP‐TF) to myeloid‐ or fibroblast‐dominated profiles (CTAP‐M, CTAP‐F, and CTAP‐EFM) and have been shown to predict differential treatment responses. We next sought to test whether the gene modules identified through network GWAS as distinguishing CCP+/RF+ and CCP−/RF+ RA endotypes also contribute to the phenotypic differences between CTAPs. To do this, we performed functional validation using scRNA‐seq data from sorted synovial cell populations. We evaluated the discriminatory power of the modules in distinguishing individual CTAPs (Figure 6C–6G). The modules exhibited CTAP‐specific discriminatory performance, with interferon signaling and ECM TGF‐β/BMP signaling modules differentiating stromal‐ and lymphocyte‐enriched CTAPs across relevant cell types (Supplementary Additional Clinical Findings).

Further, predicting patient response to specific therapeutic regimens remains a significant clinical challenge in RA. We investigated whether cell‐type–specific gene modules from our network‐based GWAS could discriminate between patients showing inadequate responses to methotrexate or TNF inhibitors (TNFi) (Supplementary Figure 3B). Indeed, the identified gene modules were discriminatory between different treatment responses across multiple cell types (Supplementary Additional Clinical Findings).

### Multivariate analysis using whole‐blood RNA‐seq data from the Pathobiology of Early Arthritis cohort validates candidate gene modules

To provide orthogonal validation of the candidate gene modules in a larger cohort, we analyzed whole‐blood RNA‐seq data from the Pathobiology of Early Arthritis^39^ cohort (n = 67). This larger data set enabled us to both validate the robustness of the prioritized modules and determine whether their signals specifically distinguished CCP+/RF+ from CCP−/RF+ RA, beyond general RF‐associated effects.

First, restricting the analysis to blood‐derived samples, we evaluated whether expression of genes from the selected modules could discriminate between patients with doubly and singly seropositive RA. We observed strong discriminator performance, particularly for module 5 (complement system activation and regulation) and module 10 (protein folding and glycosylation in the endoplasmic reticulum; Figure 6H, Supplementary Figure 3C). We then repeated the analysis comparing only RF+ and RF− patients in the same data set. We found that module 5 is specific to the contrast between doubly seropositive (CCP+/RF+) and singly seropositive (CCP−/RF+) RA (Figure 6I, Supplementary Figure 3D). Together, these analyses demonstrate that certain network modules represent very specific differences between CCP+/RF+ and CCP−/RF+, whereas others uniquely define the molecular signatures of doubly seropositive RA relative to singly seropositive RA.

## DISCUSSION

Differences in seroprevalence for autoantibodies such as CCP represent a major source of clinical heterogeneity in RA, reflecting not only phenotypic variation but distinct disease states. Multiple studies have shown that CCP+/RF+ and CCP−/RF+ RA are driven by different genetic architectures, but most of these studies were conducted using only CCP+/RF+ or CCP−/RF+ RA samples.^8^, ^9^, ^10^ Traditional GWASs have identified both shared and distinct loci across CCP+ and CCP− RA, including *ANKRD55*, *STAT4*, *C5orf30*, *PTPN22*, *ELMO1*, *RUNX1*, and *BLK*.^40^, ^41^, ^42^ Within the HLA region, *HLA‐DR4* has been associated with CCP+/RF+ RA^43^ and *HLA‐DR3* with CCP−/RF+ RA.^44^ To date, only one case‐case GWAS between CCP+/RF+ and CCP−/RF+ RA has been reported.^11^ However, due to limited sample size, this study could not identify loci beyond the HLA region. Although HLA loci contribute to phenotypic differences, they fail to explain the observed heritability gap, motivating the use of a network‐based GWAS approach to identify additional loci underlying disease heterogeneity.

Using a novel network propagation‐based approach, we identified 14 significant gene modules. Further validation using heritability partitioning and functional genomic data sets prioritized five modules, which were replicated in an independent cohort and captured additional clinical heterogeneity between the two endotypes, including CTAPs and variability in treatment response. Our systems‐level approach can partly help explain why CCP+/RF+ and CCP−/RF+ patients—although treated with the same first‐line therapies such as methotrexate or TNFi—often follow divergent therapeutic trajectories. Moreover, we observed that these endotype‐specific gene modules are not confined to the synovium; rather, some of the same modules are also detectable in peripheral blood. This makes our approach clinically valuable for patient stratification and precision medicine in RA.

These identified modules recapitulate known mechanisms of RNA pathogenesis and provide novel insights. For example, understanding the role of complement system activation in RA, particularly in the context of the complement system activation and regulation module (module 5), could open new avenues for therapeutic intervention. Targeting the components of the membrane attack complex or modulating its activity may offer a strategy to mitigate tissue damage and inflammation in RA, particularly in those with CCP+ disease.^29^, ^30^ Overall, this study demonstrates that network‐based GWAS coupled to functional genomics can help characterize interaction‐specific molecular phenotypes^45^, ^46^ linked to disease pathogenesis missed by conventional GWAS approaches.

## AUTHOR CONTRIBUTIONS

All authors contributed to at least one of the following manuscript preparation roles: conceptualization AND/OR methodology, software, investigation, formal analysis, data curation, visualization, and validation AND drafting or reviewing/editing the final draft. As corresponding author, Dr Das confirms that all authors have provided the final approval of the version to be published and takes responsibility for the affirmations regarding article submission (eg, not under consideration by another journal), the integrity of the data presented, and the statements regarding compliance with institutional review board/Declaration of Helsinki requirements.

## Supporting information


**Disclosure Form**:

**Data S1** Supplementary Notes

**Data S2:** Supplementary Additional Clinical Findings

**Data S3:** supplementary figures
