# Supplementary material for: Multimodal Integration of Protein Interactomes With Genomic and Molecular Data Discovers Distinct Rheumatoid Arthritis Endotypes
Source: Arthritis Rheumatol. 2026 Mar 9;78(8):1653–65. doi: 10.1002/art.70091 (PMC13430078; doi:10.1002/art.70091)
Supplement: Supplementary file 2 — Data S1 Supplementary Notes [file ART-78-1653-s004.pdf]

Supplementary Notes

Permutation Tests to Identify Significant Modules from Network Propagation

We generated a null distribution by randomly swapping the edges of the PPI network while preserving the degree of each node (a stringent evaluation where the random networks preserve the degree distribution of the original network), allowing us to evaluate whether observed modules (across a range of sizes) were significantly enriched beyond what would be expected by chance. The network propagation algorithm converged on high-scoring modules where individual gene scores did not necessarily meet the univariate significance threshold, but the modules overall met a stringent false discovery rate (FDR) threshold of < 0.1, based on permutation testing with degree-preserving network edge swaps. This stringent approach ensured that the identified modules reflected true topological structures in the protein network rather than artifacts of network connectivity.

Distribution-Matched Permutation Testing

These control modules were constructed by randomly sampling genes according to the distribution of node heat scores in each candidate module, deliberately disregarding the underlying protein interaction network structure. Node heat scores were categorized into three bins – high, medium, and low – to ensure the number of genes selected in each bin of the control modules matched those in the corresponding candidate modules. For example, if a gene module contained four high and one medium heat score genes, an equal number of genes were randomly selected from the respective heat bins to construct the control module. This comparison allowed us to specifically measure the contribution of the PPI network structure to the candidate modules, allowing us to assess whether the significant associations identified by the network-based method were due to the underlying biological relationships between genes, rather than random chance.

Heritability Estimation using LDAK

Unlike the uniform model, which assumes equal heritability contributions from each SNP, the LDAK model assigns weights to the expected heritability of each SNP proportional to $\mathrm{q_j}$, as presented in Equations (1,2):

$$E(h_j^2) \propto q_j \tag{1}$$

$$q_j = [p_j\,(1 - p_j)]^{0.75} \tag{2}$$

where h_j^2 represents the heritability of SNP j and q_j denotes the SNP-specific weight. $\beta_j \sim N(0, q_j\, \sigma_g{}^2)$The heritability model incorporates both fixed effects (θ) and random effects (β). In our study, sex and the first principal were considered as fixed effects, while SNP effects β_j were treated as random effects. The model defines heritability as the proportion of phenotypic variance explained by the genetic variance:

$$Y_i = W_i\theta + X_i\beta + e_i = w_{i,1}\theta_1 + \ldots + w_{i,p}\theta_p + X_{i,1}\beta_1 + \ldots + X_{i,M}\beta_m + e_i \quad (3)$$

where h^2 represents the heritability, $\sigma_y{}^2$ phenotypic variance, $\sigma_g{}^2$ is the genetic variance, and $\sigma_r{}^2$ is the residual or environmental variance $e_i \sim N(0, \sigma_r{}^2)$.

$$h^2 = \sigma_g{}^2/(\sigma_g{}^2 + \sigma_r{}^2) = \sigma_g{}^2/\sigma_y{}^2 \quad (4)$$

Network Propagation Using Random Walk With Restart Using Gene Centric Scores

HotNet2 employs a random walk with restart approach, in which the restart probability is governed by the parameter R, as shown in equation 5. Each node i in the network is assigned a vector s_i, and the elements of the transition matrix are given by:

$$s_i = R(I - (1 - R)B)^{-1}e_i \quad (5)$$

Here, e_i denotes a vector in which the i-th element is assigned a value of 1, and all other elements are set to 0. This type of vector is often referred to as a one-hot vector or a unit vector. The role of e_i is to specify the initial node from which the random walk process begins. The j-th element of the resulting vector s indicates the likelihood that a random walk starting from node i will terminate at node j after a predetermined number of steps or when the convergence condition is satisfied. To execute the HotNet2 algorithm, we input the heat scores for each gene, defined as −log (p-value) of each gene's heritability as calculated by LDAK, into the software as the initial scores for network propagation.

Heritability Partitioning Using LDAK: Module-Specific Kinship Matrix Calculation and REML Analysis

To partition heritability among different gene modules, we employed the LDAK software. The process involved calculating kinship matrices for each gene module and subsequently estimating the heritability associated with each module.

Initially, we used the LDAK command-line tool to calculate direct kinship matrices for each of the 14 predefined gene modules. The kinship matrices were calculated using the --calc-kins-direct

option, with a power parameter set to -0.25, which downweighs the contribution of SNPs with higher MAFs. The -ignore-weights YES option was applied to ensure that all SNPs contributed equally unless specified otherwise. Each module-specific list of SNPs was provided through the --extract option, and the genotype data was specified using the --bfile option, pointing to the PLINK binary file set.

Following the creation of kinship matrices, heritability partitioning was conducted using LDAK's REML (Restricted Maximum Likelihood) analysis. We specified covariates, including the first principal component and gender, through the --covar option to account for potential confounding factors. The --mgrm option was used to input a file (mgrm.list) containing the paths to the kinship matrices for all modules. The phenotype data, including the trait of interest, was provided via the --pheno option, and the specific phenotype to be analyzed was indicated using the --mpheno option.

Imputation of Gene Expression Using PrediXcan for Tissue-Specific Analysis of Discriminatory Network Modules

In our study, we employed the PrediXcan method to impute gene expression levels from genotype data, focusing on tissue-specific models relevant to rheumatoid arthritis (RA) endotypes. PrediXcan uses a linear model to predict gene expression based on learnt allele-expressions relationships, where $w_{g,k}y_g$ is the expression trait for gene g, and X_k is the number of reference allele for marker k and $w_{g,k}$ the contribution of each X_k to the expression trait. This model allows for the imputation of gene expression levels purely based on genetic variation.

For our analysis, we imputed gene expression data across various tissue types, including whole blood and subcutaneous adipose tissue. These tissues were selected due to their relevance in RA pathogenesis. By applying PrediXcan, we were able to assess whether genes within specific network modules displayed distinct expression profiles across these tissue types, enabling us to identify gene networks that may be discriminatory between different RA endotypes.

After the imputation, we performed linear regression analyses to evaluate the association between imputed gene expression and the phenotypic trait of interest. This was done by merging the imputed expression data with phenotype data and fitting linear models (lm function from base r package) to examine the relationship between gene expression and two RA endotypes. The use of a linear model allowed us to quantify the performance of tissue specific network gene modules

associated with RA endotypes. We use -log(p-value) of the regression model to quantify the significance of the modules in tissue-specific discrimination between CCP+ and CCP- RA.

Evaluation of the Discriminatory Performance of Gene Modules Using Bulk RNA-seq Data

Bulk RNA-seq data and metadata from AMP RA phase 1 study were retrieved from Immport (study accession code SDY998). Bulk RNA-seq data corresponded to sorted T cells, B cells, monocytes, and fibroblasts from disaggregated synovial tissues. The methods for data collection are described in the source paper (6). CCP and RF serostatus information was available for 29 patients, including 19 CCP^{+}/RF^{+} individuals and 10 patients who were either singly positive (CCP^{-}/RF^{+} or CCP^{+}/RF^{-}) or double negative (CCP^{-}/RF^{-}). For each cell type, genes with expression variability below the 25th percentile across the dataset were removed prior to analysis. To evaluate the discriminatory power of gene modules identified through prior network-based analyses, we applied logistic regression with L1 regularization. Analyses were conducted in R using multiple packages to support data preprocessing, model fitting, and parallel computation. Ensembl gene identifiers were mapped to gene symbols using the biomaRt package, while parallelization was implemented using doParallel and foreach. Model training and evaluation were performed using the caret framework, with LASSO-regularized logistic regression implemented via the glmnet method. Model performance was assessed using leave-one-out cross-validation, and a custom training control incorporating area under the ROC curve (AUC) was used to select optimal tuning parameters (lambda and alpha).

Replication GWAS Using the All of Us Cohort

As a replication cohort, we analyzed 248 participants from the All of Us Research Program (20) . Participants were filtered to include only Caucasian individuals who were RF+ (RF > 20 IU/mL). These individuals were further stratified based on CCP levels into the two endotypes: CCP+/RF+ (n = 93) and CCP-−/RF+ (n = 155). This study was approved by the All of Us Institutional Review Board.

Phenotypic data for each subject was represented by a vector Y with 248 entries, where a value of 1 corresponded to CCP+/RF+ status and 0 to CCP-/RF+ status. Genotype data for each individual, covering 6,879,629 SNPs, was organized into a 248 × 6,879,629 matrix X—each row represented an individual's genotype, and each column corresponded to a specific SNP. Quality control, including a genotype missingness filter of 10% (--geno 0.1) to exclude SNPs with a high proportion of missing data and a MAF threshold of 5% (--maf 0.05) to exclude rare variants, was conducted using PLINK.

Discriminatory Power of Gene Modules in Classifying CTAPs and Treatment Responses

To evaluate the predictive capacity of the gene modules in other functional contexts, we implemented a logistic regression modeling framework using scRNA-seq data from 70 RA patients (7). The dataset consisted of gene expression matrices for multiple immune and stromal cell types, along with metadata annotated with CTAP labels or clinical treatment response categories. For each cell type, we first generated pseudo-bulk expression profiles by aggregating single-cell counts across cells from the same patient.

Using these pseudo-bulk profiles, we constructed binary classification models based on genes from the identified modules to distinguish a given CTAP or treatment response category from all others in a one-vs-rest framework. Model training was performed using L1-penalized logistic regression (LASSO) implemented via the glmnet package in R. Five-fold cross-validation was applied for CTAP classification tasks, while 10-fold cross-validation was used for treatment response prediction.

All modeling steps, including training, hyperparameter tuning, and performance evaluation, were coordinated using the caret package. Discriminatory performance was assessed using receiver operating characteristic (ROC) curves generated with the pROC package, with area under the curve (AUC) serving as the primary evaluation metric across CTAPs, treatment categories, and cell types. Gene expression filtering and data preprocessing were conducted using base R and the dplyr package.
