# Supplementary material for: Multimodal Integration of Protein Interactomes With Genomic and Molecular Data Discovers Distinct Rheumatoid Arthritis Endotypes
Source: Arthritis Rheumatol. 2026 Mar 9;78(8):1653–65. doi: 10.1002/art.70091 (PMC13430078; doi:10.1002/art.70091)
Supplement: Supplementary file 3 — Data S2: Supplementary Additional Clinical Findings [file ART-78-1653-s003.pdf]

Supplementary Additional Clinical Findings

Gene Modules Stratify by CTAPs

CTAP-EFM, characterized by an enrichment of endothelial cells, fibroblasts, and myeloid cells, is associated with a comparatively less inflammatory microenvironment than the lymphocyte-rich CTAP subtypes and reflects a transitional or remodeling state of the synovium (7). Of our candidate gene modules, gene expression models for the TGF-beta/BMP signaling in developmental processes (module 2) and interferon signaling and ECM modules (module 1) showed the best discriminatory performance for CTAP-EFM (Figure 6C). These modules were also able to discriminate CTAP-F, which shows an enrichment for fibroblasts, from other CTAPs (Figure 6D). Quantitatively, in CTAP-EFM, the myeloid cell type showed strong discrimination between double and single seropositive RA with an AUC of 0.77, while the fibroblast cell type (module 1) achieved an identical AUC of 0.77. In CTAP-M, module 1 in myeloid cells reached an AUC of 0.74 and module 2 in fibroblasts reached an AUC of 0.74, reflecting comparable predictive strength across stromal compartments.

The ability of these modules to distinguish CTAPs that are both stromal-enriched and characterized by lower levels of inflammation underscores the functional relevance of the underlying molecular programs captured by these modules. These gene modules represent key processes associated with inflammatory microenvironments and ECM remodeling, highlighting their relevance not only to seroprevalence of autoantibodies but also to broader clinical heterogeneity observed in RA.

CTAP-M, the myeloid-enriched cell-type abundance phenotype, is characterized by a high abundance of monocytes and macrophages, with relatively low infiltration of lymphocytes. All four gene modules showed good discriminatory performance for this phenotype in myeloid cells (Figure 6E). Notably, CTAP-M was found to be associated with CCP-negative status (7). While the modules do capture underlying differences in serological status, their strong discriminatory capacity in CTAP-M suggests that they extend beyond serostatus alone. Their performance likely reflects sensitivity to additional biological processes, such as myeloid-driven inflammation, that define this phenotype and are not solely explained by autoantibody levels. Consistent with this interpretation, in CTAP-M the myeloid module 2 yielded an AUC of 0.70, whereas fibroblast module 2 showed a higher AUC of 0.83, indicating particularly robust separation within stromal lineages.

CTAP-TB and CTAP-TF represent lymphocyte-enriched phenotypes, marked by high infiltration of lymphocytes within the synovium. Both these phenotypes were more commonly seen in patients with CCP positive synovium, which has been associated with higher lymphocyte infiltration compared to CCP negative synovium (7). Both CTAP-TB and CTAP-TF exhibit transcriptional signatures of adaptive immune activation, including elevated expression of T follicular helper cell markers, immunoglobulin genes, and cytokines such as IL-21, consistent with the presence of functionally active T–B cell interactions. Notably, the interferon signaling and ECM module (module 1) exhibited strong discriminatory power for these CTAPs (Figure 6F-G), highlighting the role of these gene modules in adaptive immune–driven inflammation. Additionally, the good performance of the TGF-beta/BMP signaling in developmental processes module (module 2) across the two phenotypes suggests that TGF-beta signaling may contribute to the regulation of lymphocyte activity and potentially influence B cell activation and autoantibody production in these CTAPs. Specifically, in CTAP-TB, module 1 achieved an AUC of 0.80 in T cells and module 2 achieved an AUC of 0.75 in B cells, underscoring their ability to delineate lymphocyte-enriched microenvironments.

Gene Modules Stratify by Responsiveness to Therapy in RA

The T cell-associated gene module 1 was significantly predictive of inadequate TNFi responses. Moreover, the modules linked to myeloid cells, B cells, and T cells displayed predictive capability for methotrexate inadequacy. Several modules exhibited strong predictive value for non-response to methotrexate (MTX) and TNF inhibitors (TNFi) (Supplementary Fig. 3B). For instance, module 1, characterized by type I interferon signaling and ECM and predominantly active in T cells and fibroblasts, was enriched among TNFi non-responders. This suggests a disease process driven by interferon activity and fibroblast remodeling that may not be adequately controlled by TNF blockade alone, potentially requiring alternative or combination therapies. MTX non-response, in contrast, was associated with the combined activation of modules 1, 2, 5, and 10 across myeloid, B, and T cells, encompassing TGF-beta/BMP signaling, complement activation, and cellular stress in protein-folding pathways. Together, these patterns indicate that different therapeutic responses may arise when disease activity is dominated by biological processes not effectively targeted by standard MTX or TNFi treatments. (Supplementary Figure 3B) and suggest that the identified modules are functionally relevant and capture heterogeneity beyond the seroprevalence of autoantibodies in RA.
