# Supplementary figures and images for: Multimodal Integration of Protein Interactomes With Genomic and Molecular Data Discovers Distinct Rheumatoid Arthritis Endotypes
Source: Arthritis Rheumatol. 2026 Mar 9;78(8):1653–65. doi: 10.1002/art.70091 (PMC13430078; doi:10.1002/art.70091)

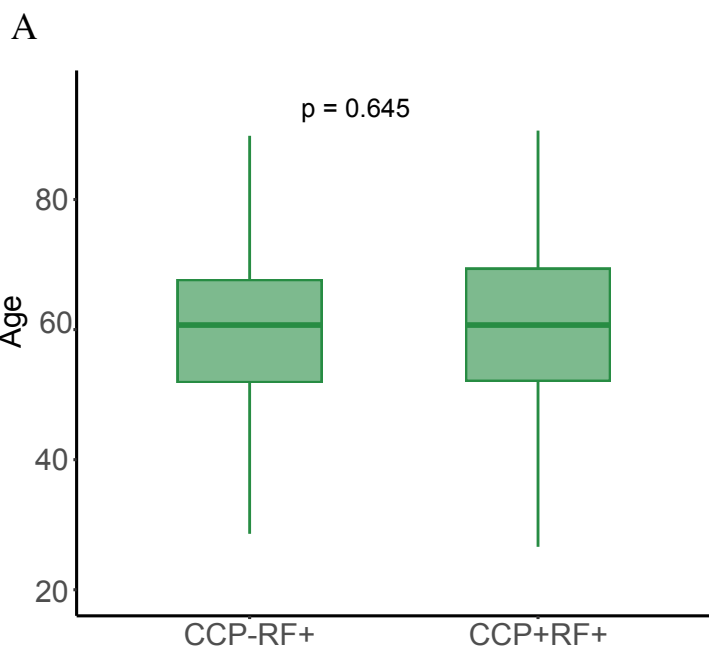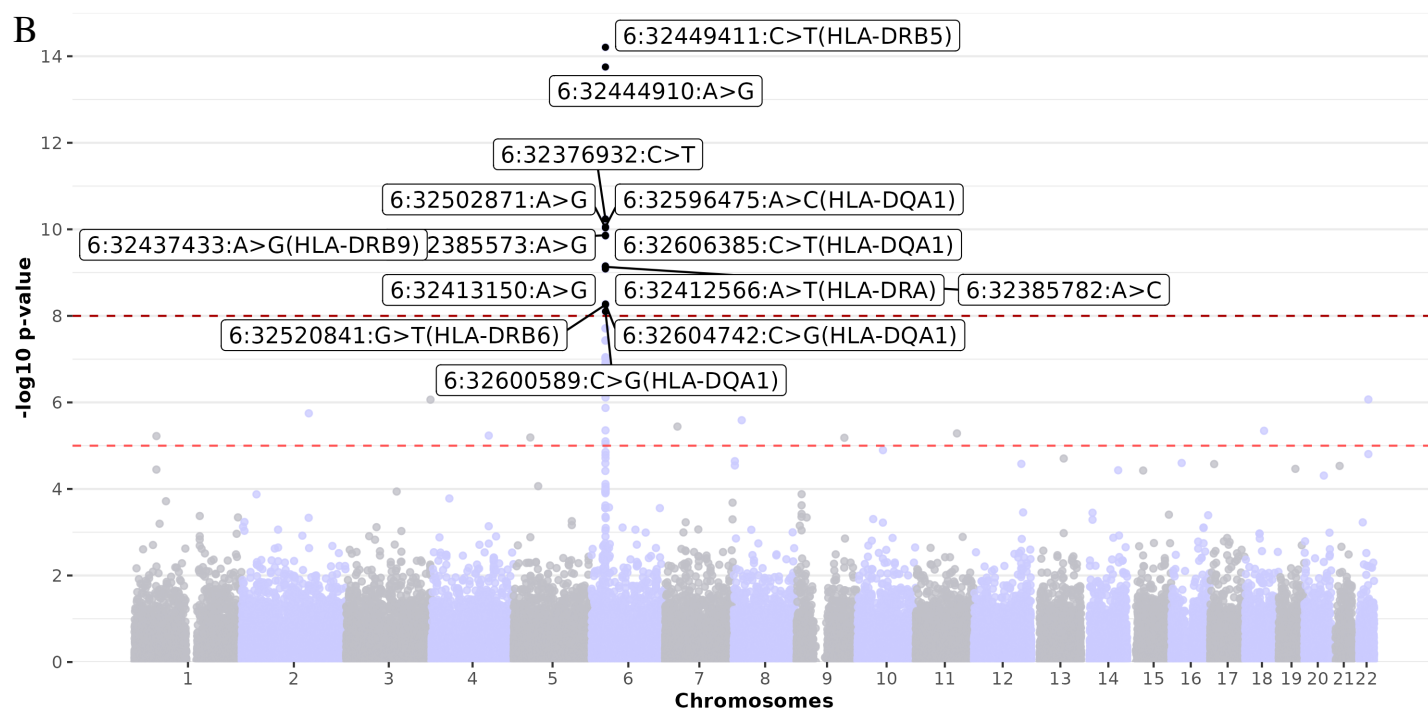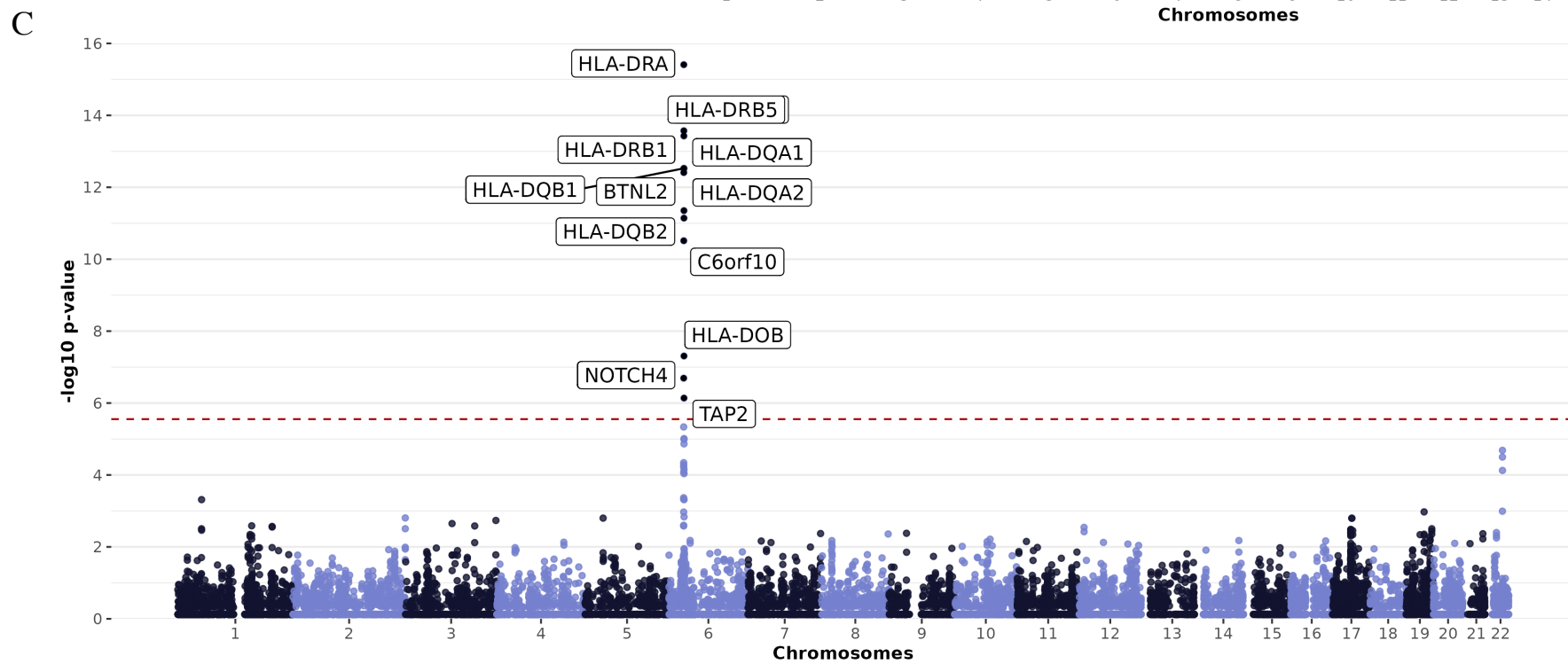

Module 3

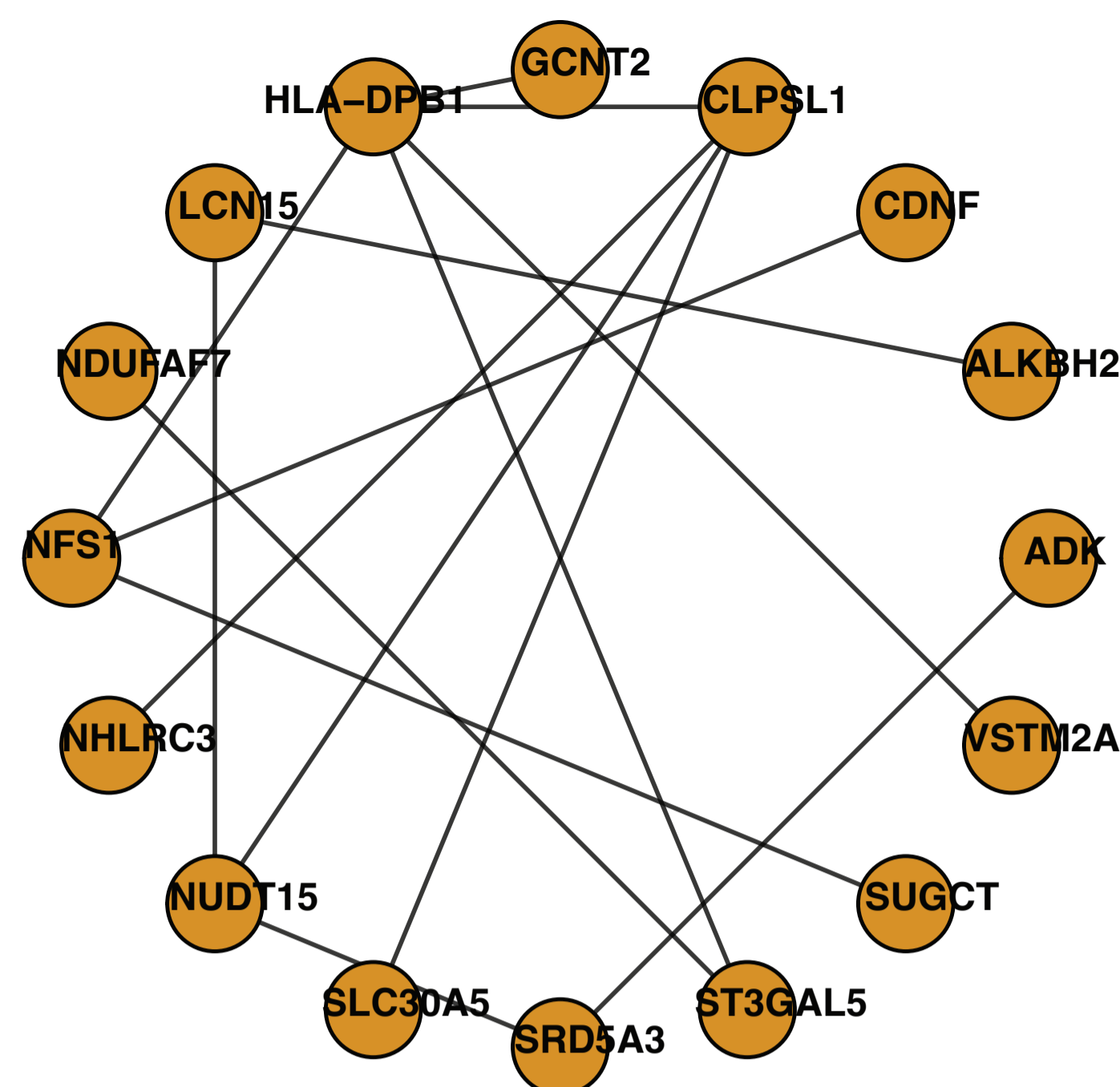

Module 4

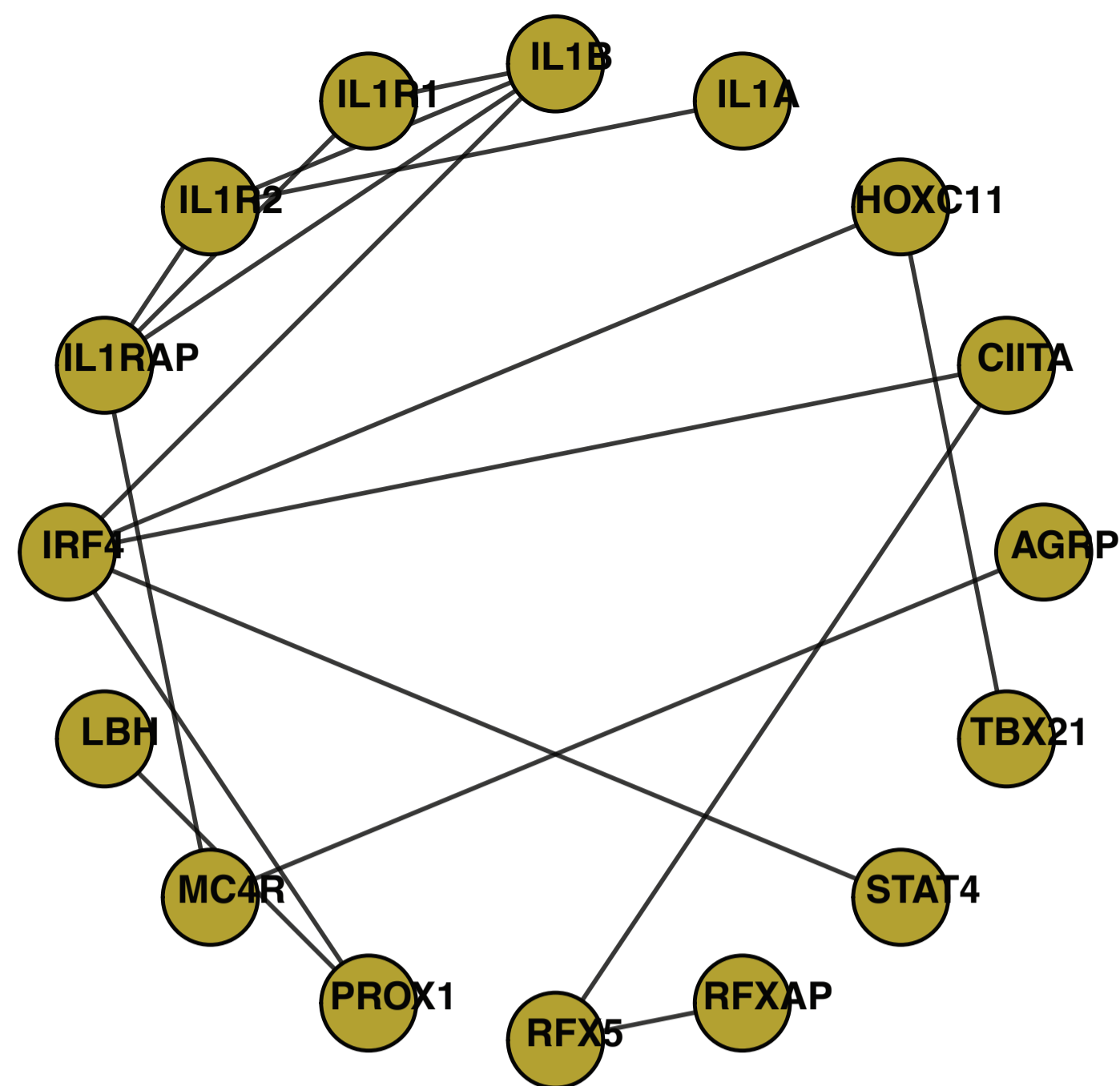

Module 6

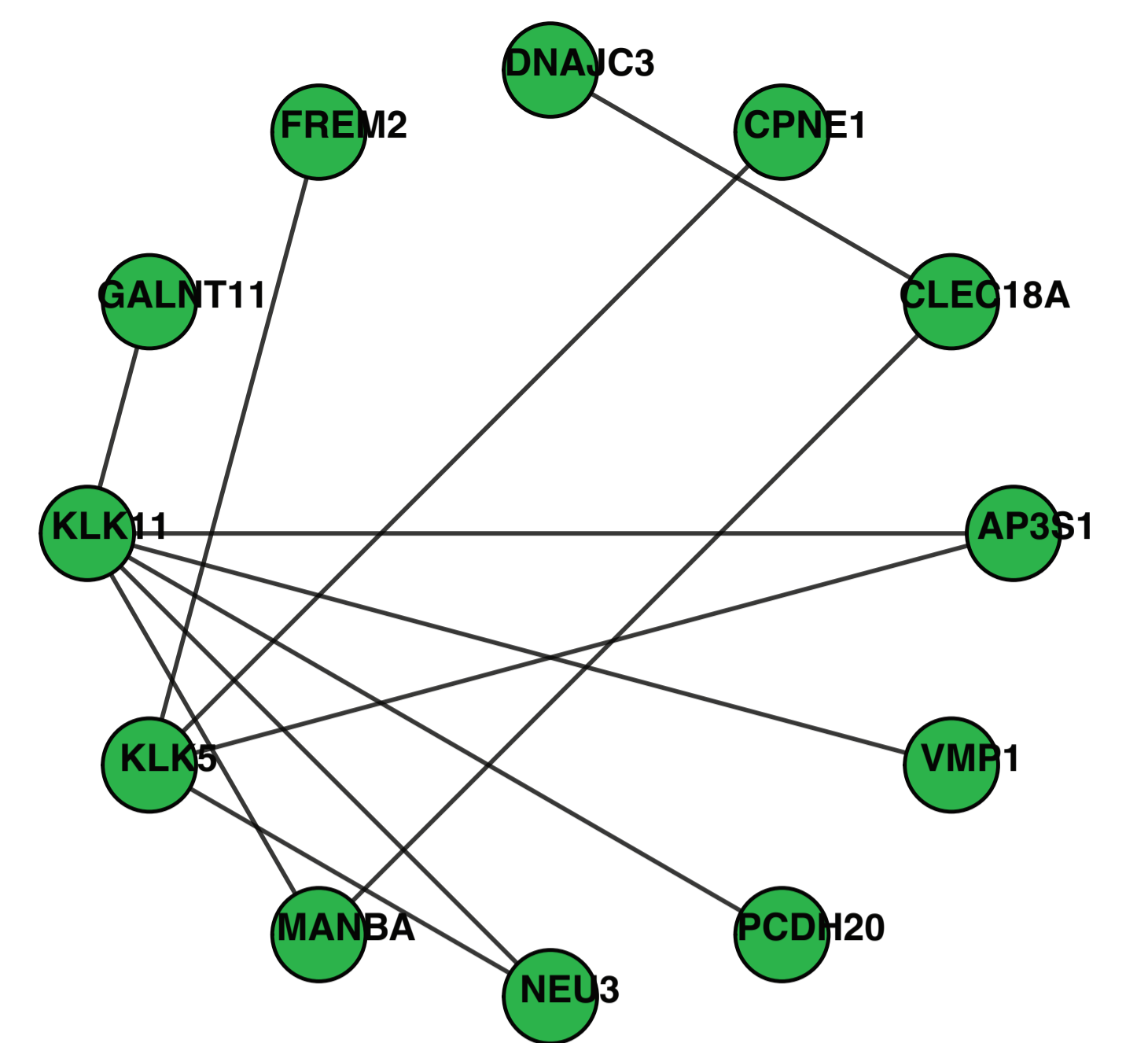

Module 7

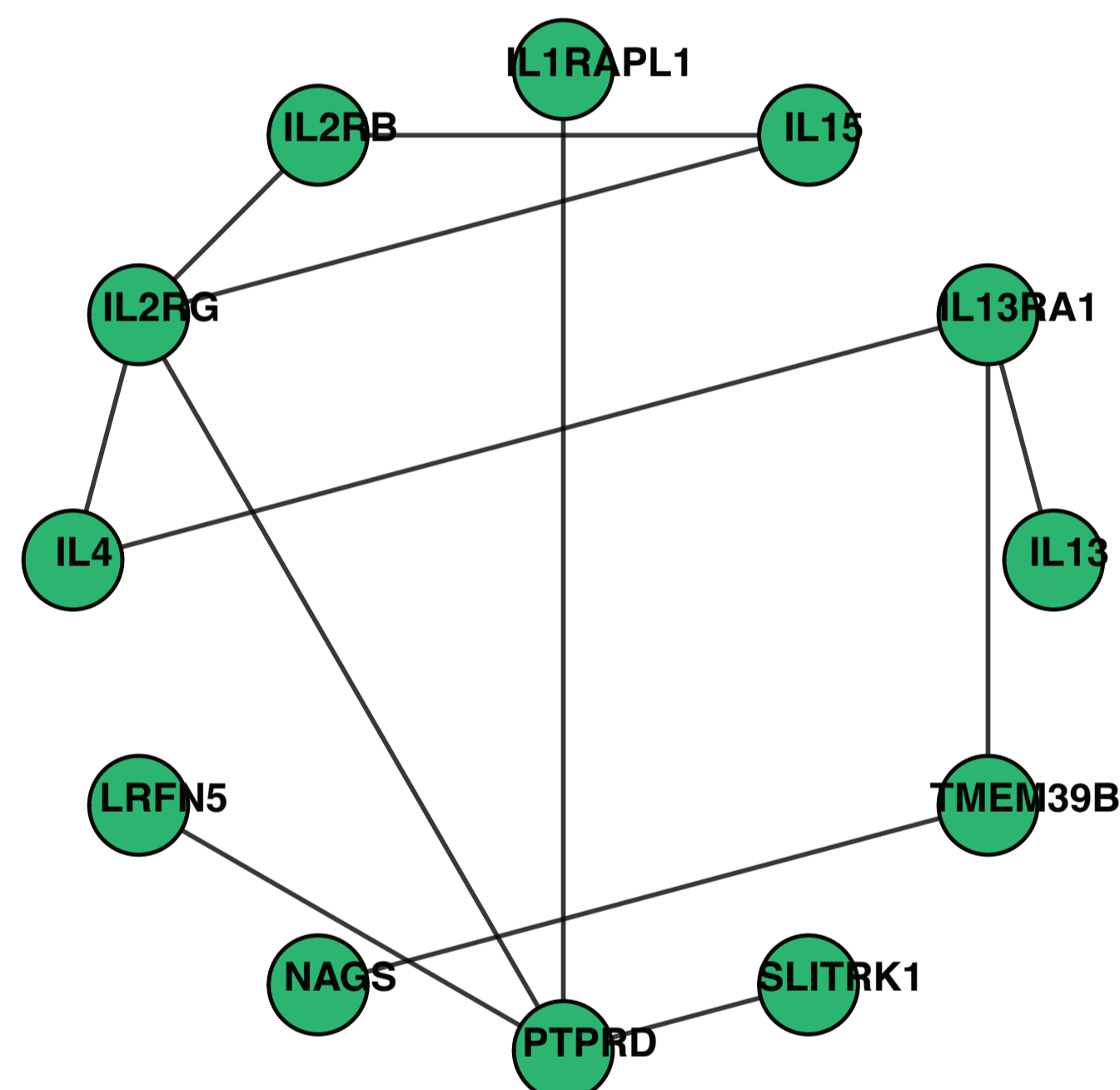

Module 8

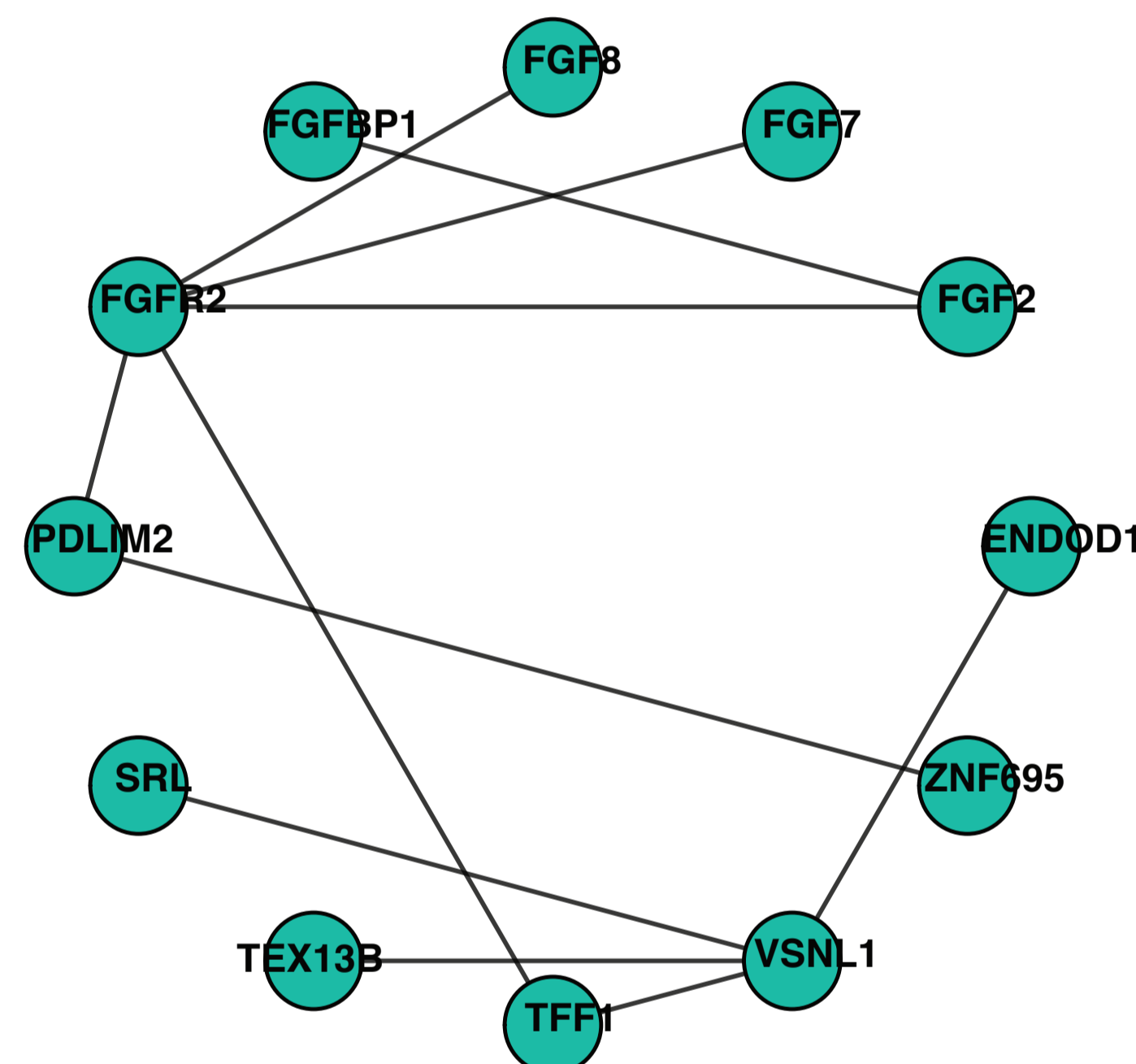

Module 9

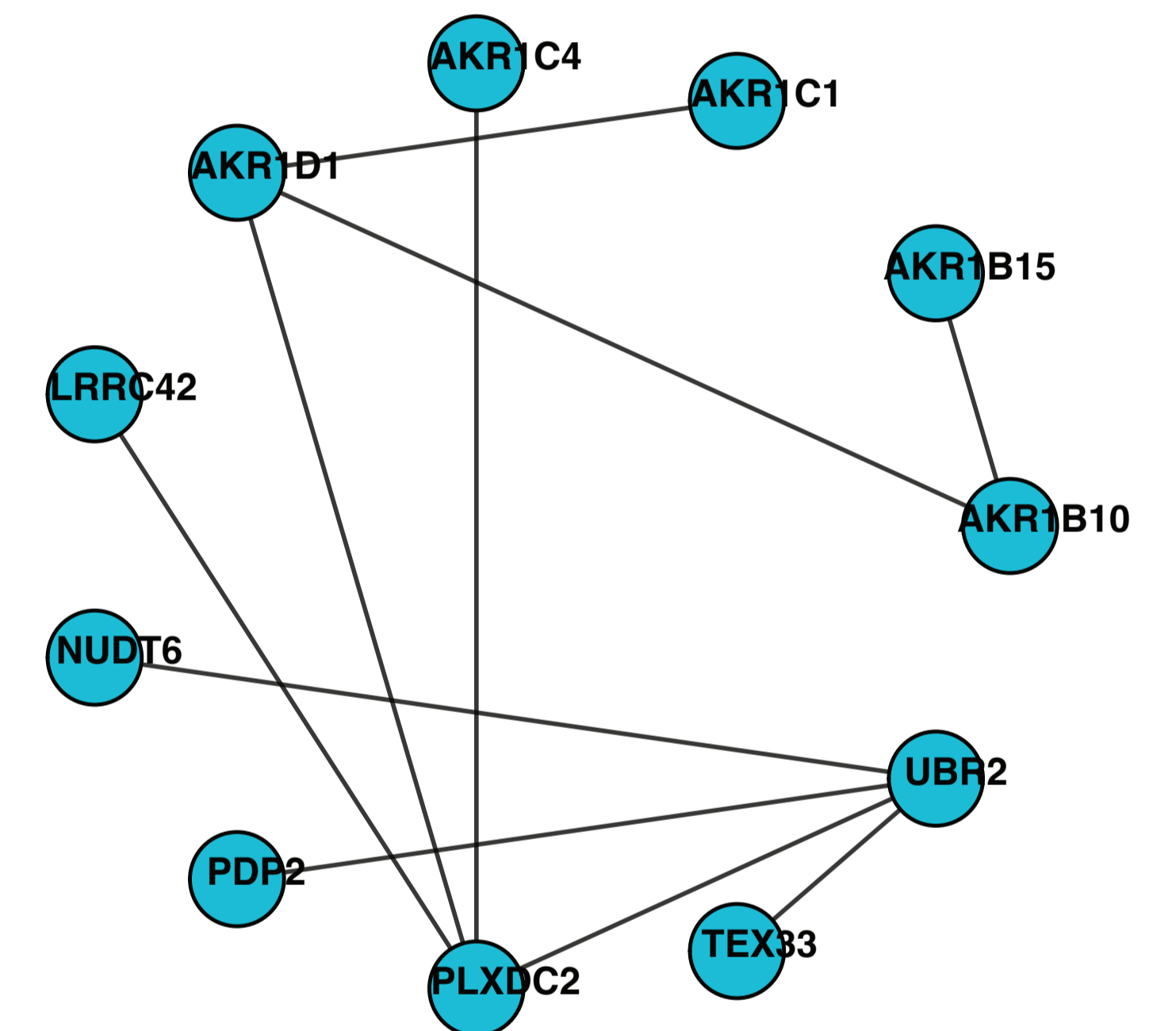

Module 11

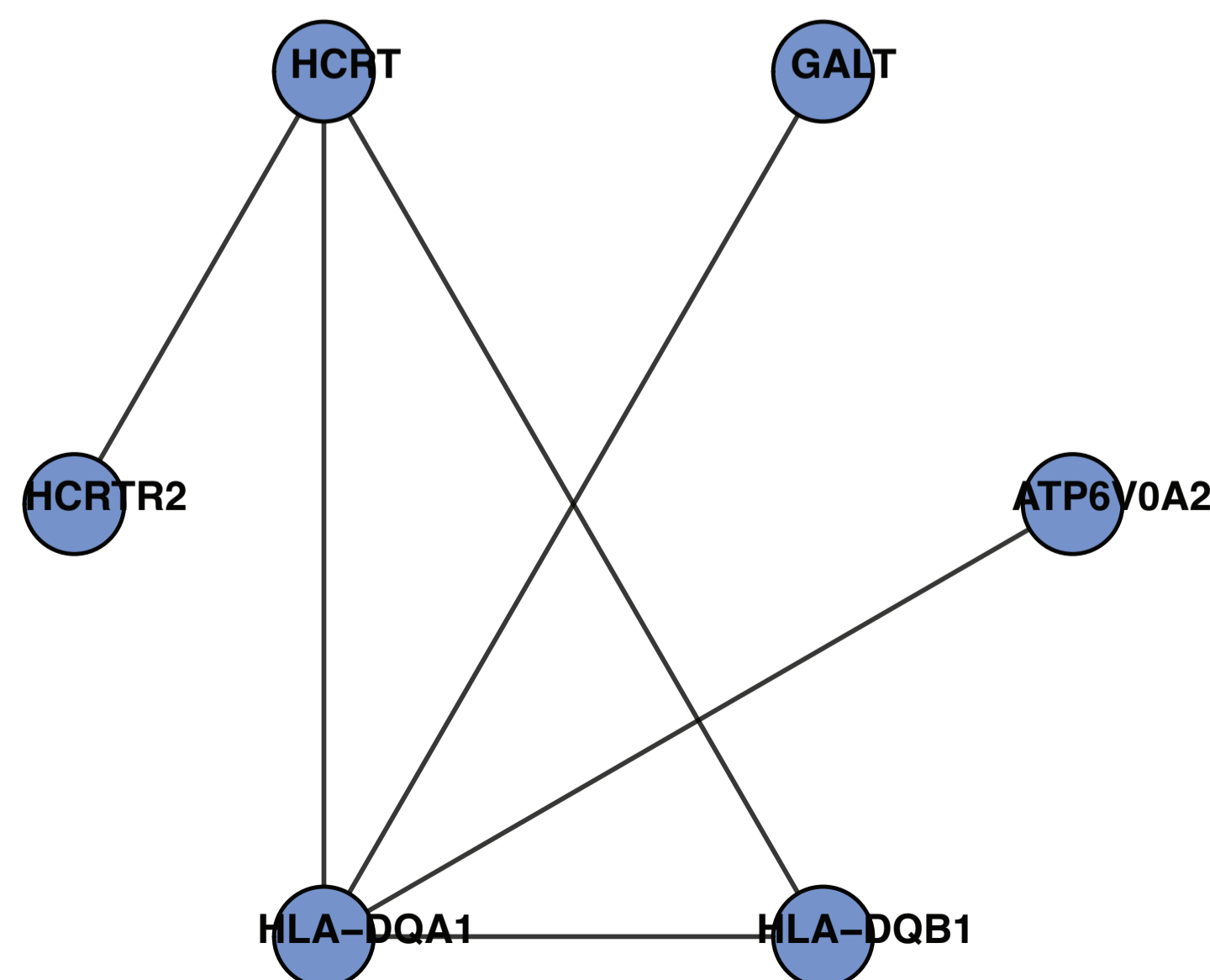

Module 12

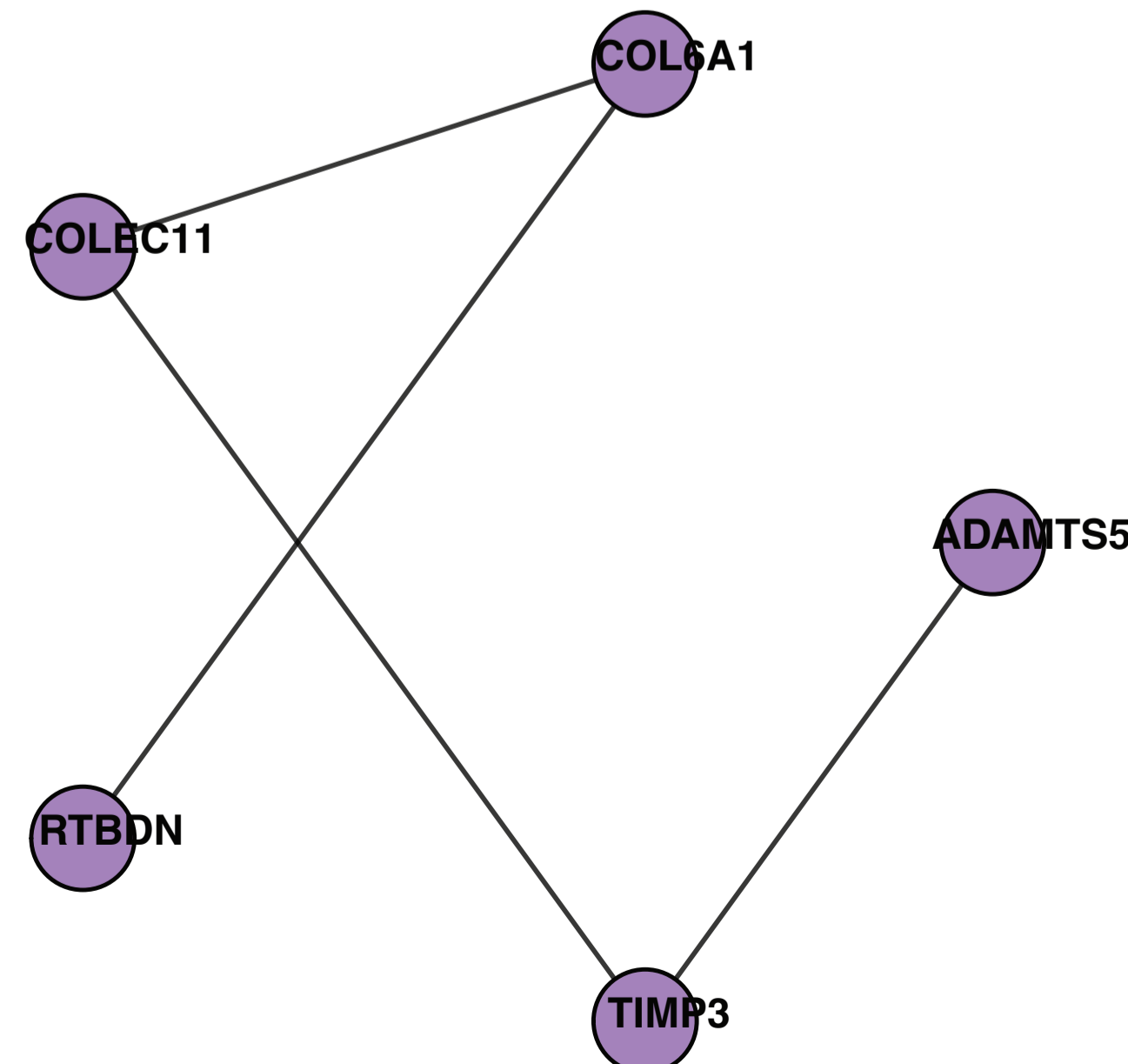

Module 13

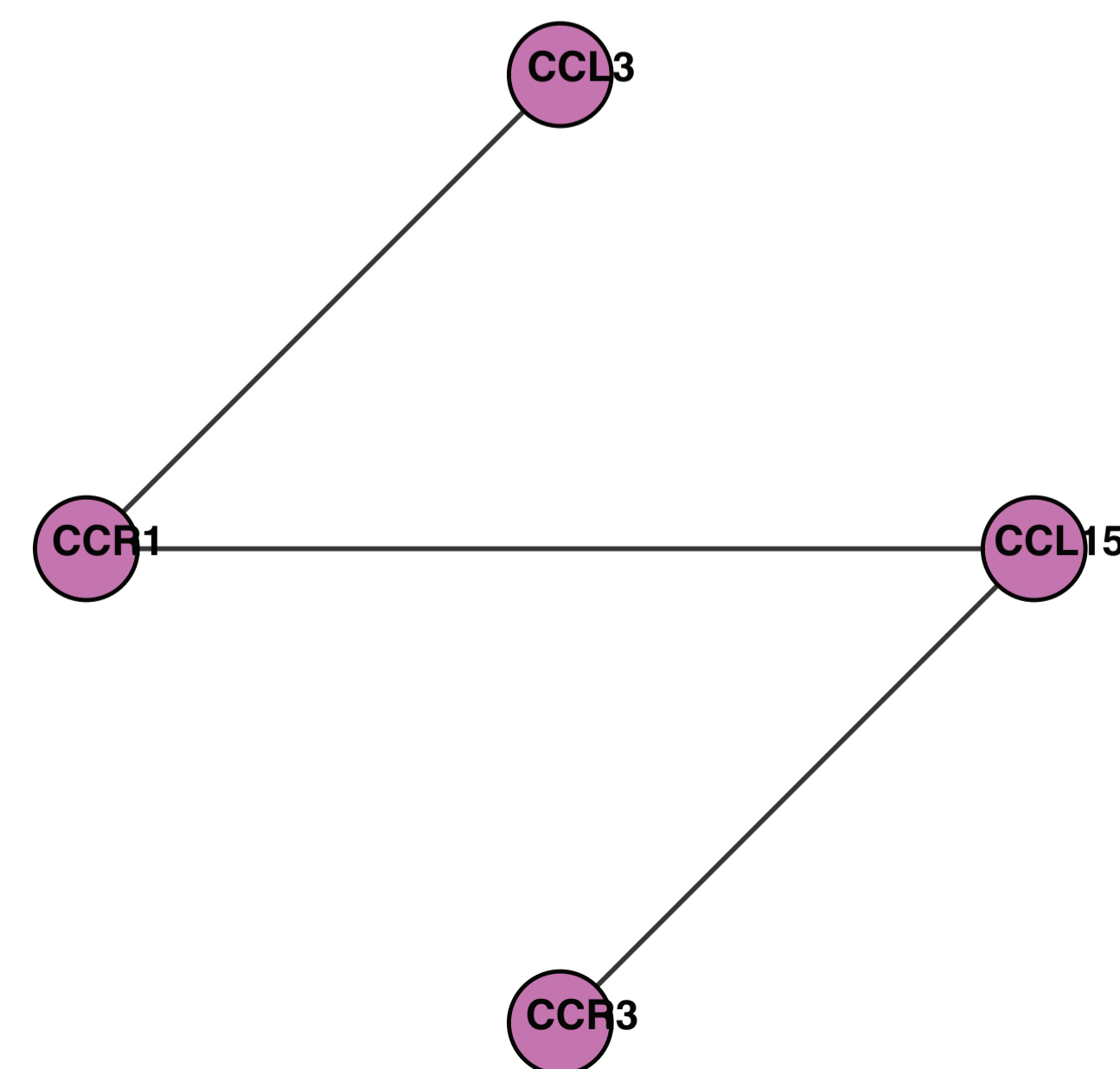

Module 14

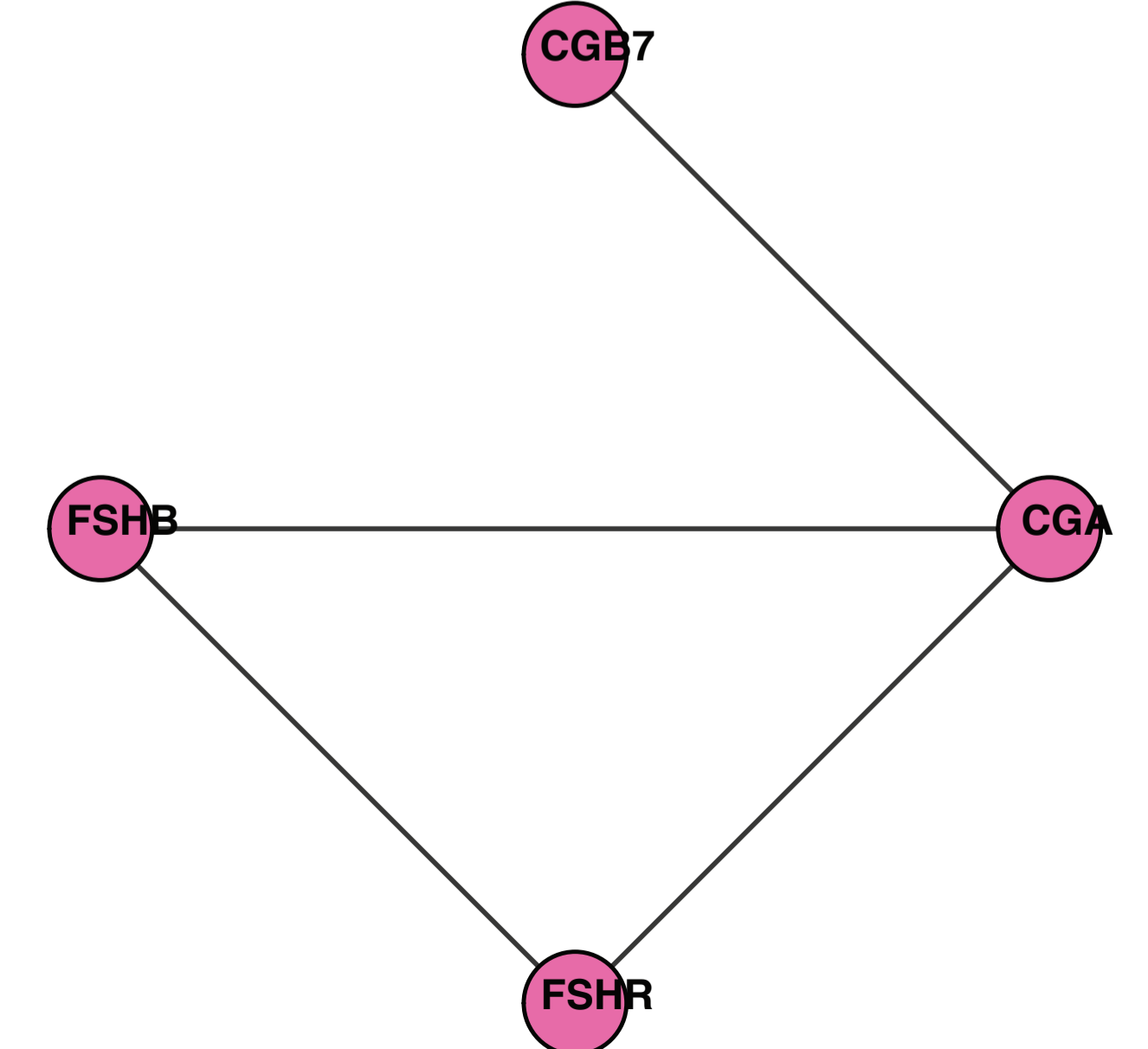

A

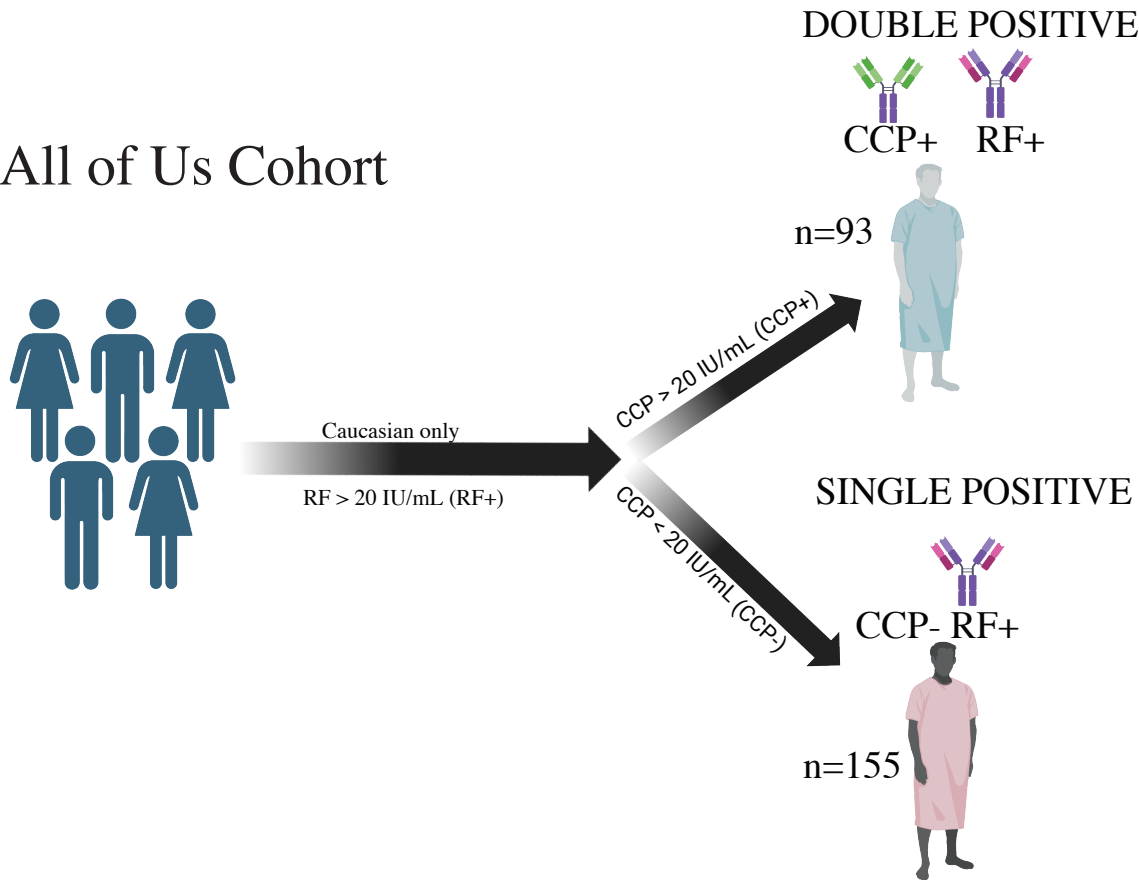

B

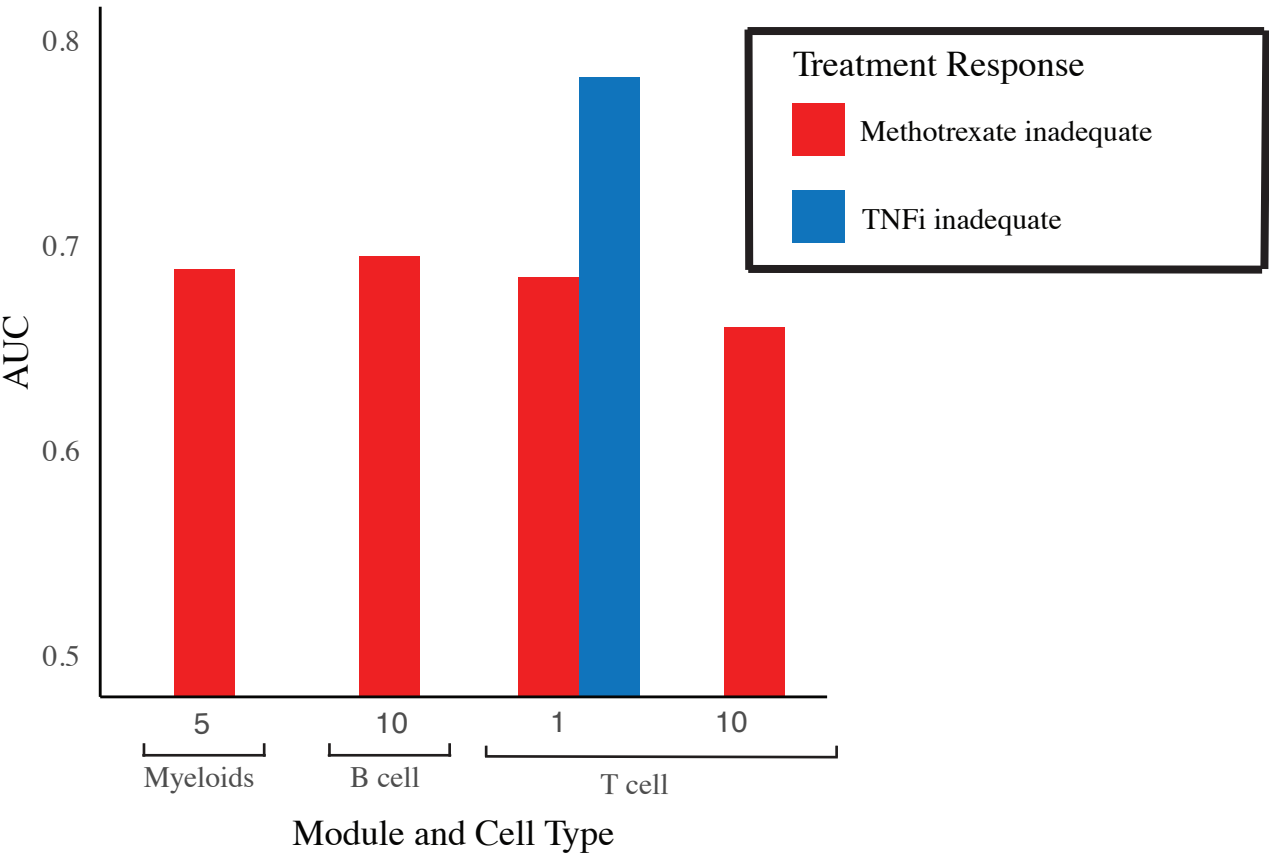

C

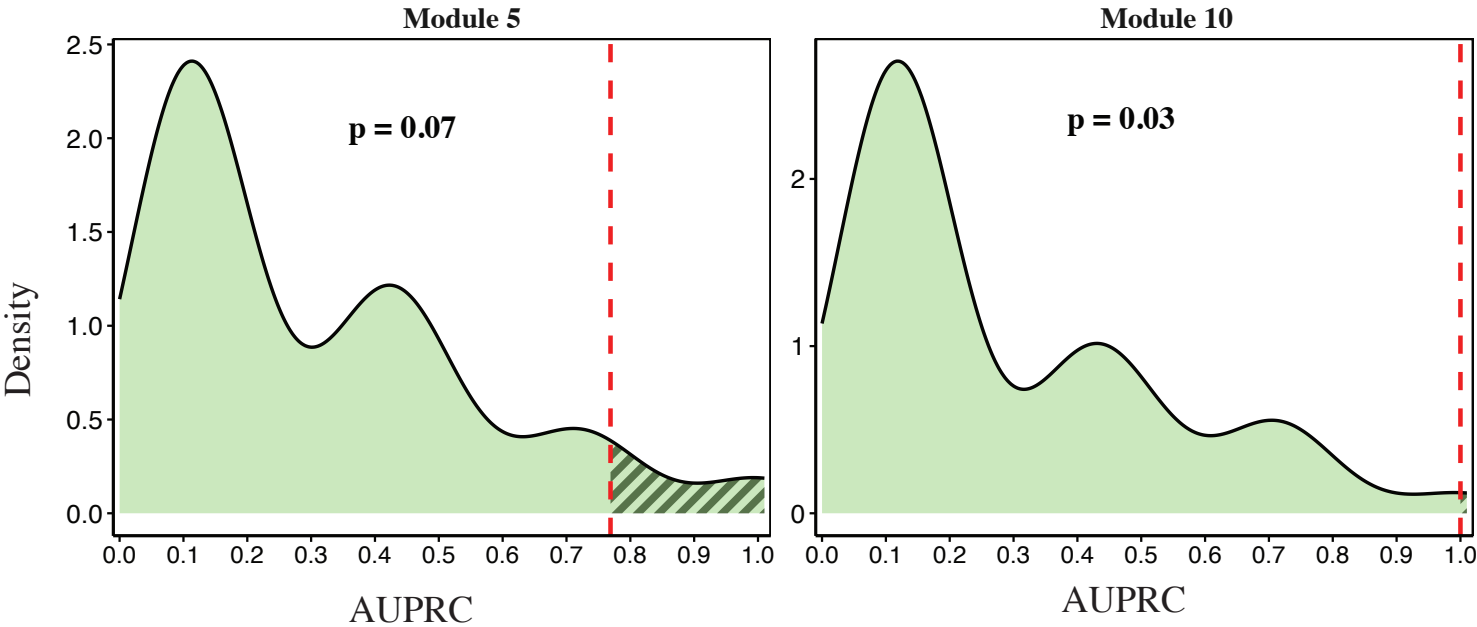

D

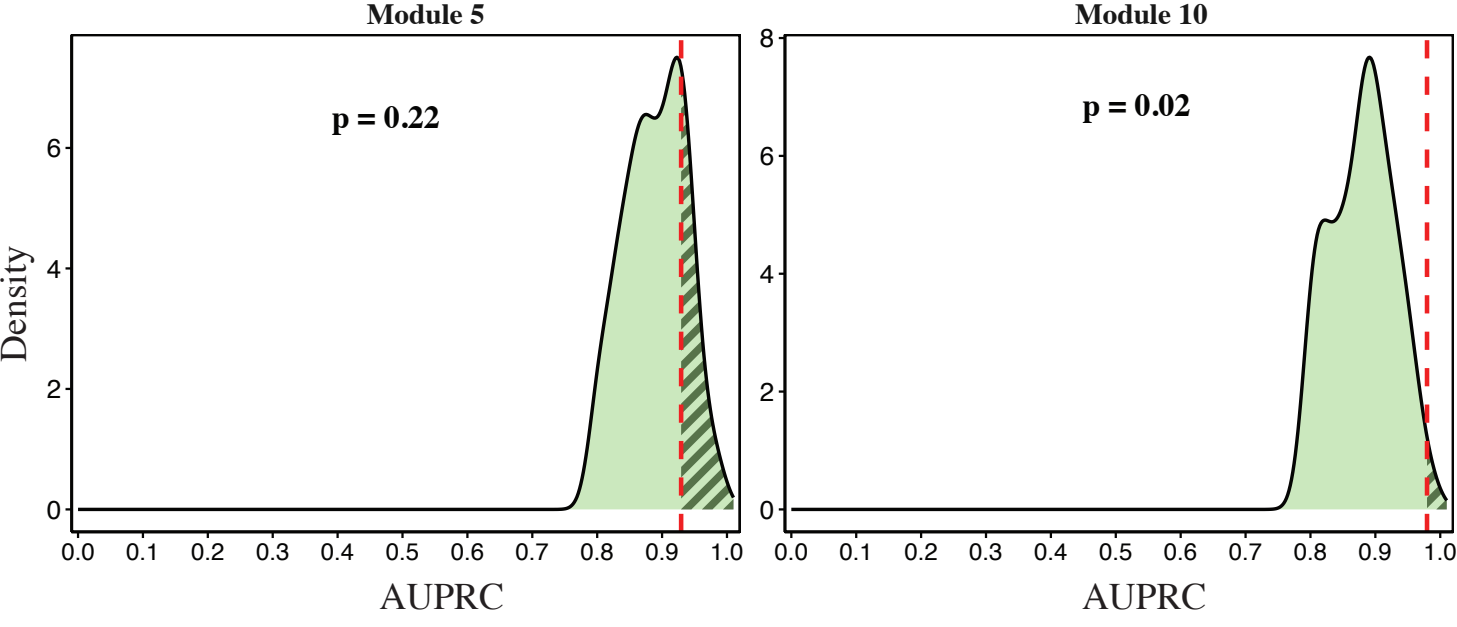

Supplement: Supplementary file 4 — Data S3: supplementary figures [file ART-78-1653-s002.pdf]
